# Therapeutic drug monitoring of zonisamide in children with epilepsy: development and validation of a precise binary prediction model

**DOI:** 10.3389/fphar.2026.1841966

**Published:** 2026-08-27

**Authors:** Huiying Li, Xiaoling Tang, Fashuang Li, Jing Zong, Yunwei Li, Linbo Li

**Affiliations:** 1 Department of Pharmacy, Kunming Children’s Hospital, Kunming, China; 2 Department of Orthopedics, Kunming First People’s Hospital, Kunming, China

**Keywords:** concentration, individualized dosing, machine learning, pediatric epilepsy, zonisamide

## Abstract

**Objective:**

This study aimed to investigate the key factors influencing the failure to achieve steady-state trough concentrations of zonisamide in pediatric patients with epilepsy. Furthermore, we sought to construct and validate a binary machine learning prediction model for zonisamide concentration attainment to provide an objective reference for therapeutic drug monitoring in clinical practice.

**Methods:**

A retrospective analysis was conducted on 958 pediatric patients with epilepsy who received zonisamide treatment at the Department of Neurology, Kunming Children’s Hospital, from May 2022 to January 2026. Multi-dimensional feature screening was performed on the training set using spearman correlation analysis, variance inflation factor multicollinearity diagnosis, univariate analysis, lasso regression, boruta algorithm, and 500-iteration bootstrap resampling. Subsequently, 17 machine learning classification models were developed. Hyperparameter optimization was achieved through grid search combined with 10-fold cross-validation. Model performance was comprehensively evaluated in terms of discrimination, calibration, and clinical net benefit using 500-iteration bootstrap resampling. Finally, SHAPley additive exPlanations analysis and a visualized decision tree were employed to interpret the feature contribution and intrinsic decision logic of the optimal model.

**Results:**

Following multi-stage screening, six core predictive variables were identified: dosage, uric acid, age, total bilirubin, vitamin D, aspartate aminotransferase, and gender. A comparative analysis of the 17 machine learning models revealed that the classification and regression tree model exhibited the best comprehensive predictive performance, demonstrating superior stability and robustness under resampling validation. The area under the curve values for the internal test set and the time-split internal validation set were 0.82 and 0.87, respectively. Calibration curves and decision curve analysis confirmed ideal calibration and a wide range of clinical net benefit. SHAPley additive exPlanations analysis and decision tree results confirmed that dosage is the primary determinant of the probability of achieving target ZNS concentrations. Sex, liver and kidney function, and nutrition-related indices collectively modulate the processes of drug absorption, metabolism, and clearance, thereby influencing the risk of failing to achieve target concentrations.

**Conclusion:**

The machine learning prediction model constructed using the CART algorithm can accurately and stably predict whether steady-state trough concentrations of zonisamide reach the target therapeutic range in pediatric patients with epilepsy.

## 1 Introduction

Epilepsy represents one of the most prevalent chronic neurological disorders in childhood, characterized by high incidence rates, complex etiologies, and prolonged treatment durations (GBD Epilepsy Collaborators, 2025; Gülegen et al., 2025; Coppola et al., 2009). Pharmacotherapy remains the primary approach for seizure control. As a novel antiepileptic drug, zonisamide (ZNS) exerts its therapeutic effects by inhibiting neuronal hyperexcitability through blockade of voltage-dependent sodium channels and reduction of T-type calcium currents (Wu et al., 2026; Bobylova and Borovikov, 2025). This agent demonstrates notable efficacy in managing pediatric refractory epilepsy, particularly in cases of infantile spasms and Lennox-Gastaut syndrome. However, ZNS pharmacokinetics in children exhibit marked age-dependent and nonlinear characteristics (Mgidal et al., 2024). Studies indicate that clearance rates of ZNS are significantly higher in younger children (0–4 years) compared to older patients with epilepsy and adults (Wallander et al., 2014). Furthermore, concomitant administration with enzyme-inducing antiepileptic drugs (e.g., carbamazepine, phenytoin) accelerates ZNS metabolism, leading to substantially reduced plasma concentrations (Sills and Brodie, 2007). Such interindividual variability necessitates tailored dosing regimens, underscoring the importance of precision medicine in optimizing ZNS therapy for pediatric patients with epilepsy.

Therapeutic drug monitoring (TDM) serves as a pivotal technique for individualized dosing of anti-seizure drugs. For ZNS, the therapeutic range of 10.00–40.00 μg/mL demonstrates optimal efficacy with favorable tolerability. Subtherapeutic concentrations (<10.00 μg/mL) may result in inadequate seizure control, while supratherapeutic levels (>40.00 μg/mL) are associated with adverse effects including psychiatric symptoms, nephrolithiasis, and decreased appetite (Nersesjan et al., 2025). Routine concentration monitoring enables clinicians to identify suboptimal dosing and make timely adjustments. However, conventional TDM approaches exhibit inherent limitations due to their retrospective nature, requiring steady-state sampling before dose optimization can be implemented. This “feedback-based” adjustment proves particularly inadequate when managing pediatric patients with rapidly evolving conditions, necessitating the development of predictive tools for prospective concentration estimation during the early treatment phase.

Recent years have witnessed remarkable advancements in machine learning applications within the medical field. In TDM, various algorithms have been employed to construct predictive models. Studies on antiepileptic drugs have demonstrated that random forest (RF) and eXtreme gradient boosting (XGBoost) models outperform traditional linear regression in predicting carbamazepine concentrations (Huang et al., 2025). Similarly, machine learning has shown superior accuracy in forecasting adverse drug reactions for levetiracetam (Zhu et al., 2026), highlighting its potential in uncovering clinical data patterns and predicting therapeutic outcomes. Nevertheless, research on pediatric ZNS monitoring remains limited by several critical gaps: (1) existing studies rely on small sample sizes and cross-sectional designs, lacking large-scale longitudinal data to ensure model robustness. (2) most investigations still employ simplistic linear regression, failing to leverage machine learning’s capacity for handling nonlinear relationships and high-dimensional interactions. (3) model validation predominantly depends on internal cross-validation without external cohort verification, hindering clinical translation. (4) current models primarily focus on continuous concentration prediction rather than binary classification of “target attainment,” which holds greater clinical relevance for ZNS.

To address these limitations, this study utilizes a large-scale retrospective clinical dataset to systematically identify key determinants of ZNS target attainment through correlation analysis, collinearity diagnostics, univariate screening, lasso regression, and random forest-based feature selection. Multiple machine learning-based binary classification models will be developed and rigorously evaluated via internal cross-validation and the time-split internal validation (TSIV) cohort validation. The ultimate goal is to establish a clinically applicable predictive tool for ZNS concentration target attainment in pediatric patients, thereby facilitating evidence-based individualized dosing and advancing precision medicine in childhood epilepsy treatment.

## 2 Materials and methods

### 2.1 Study population

**FIGURE 1:**
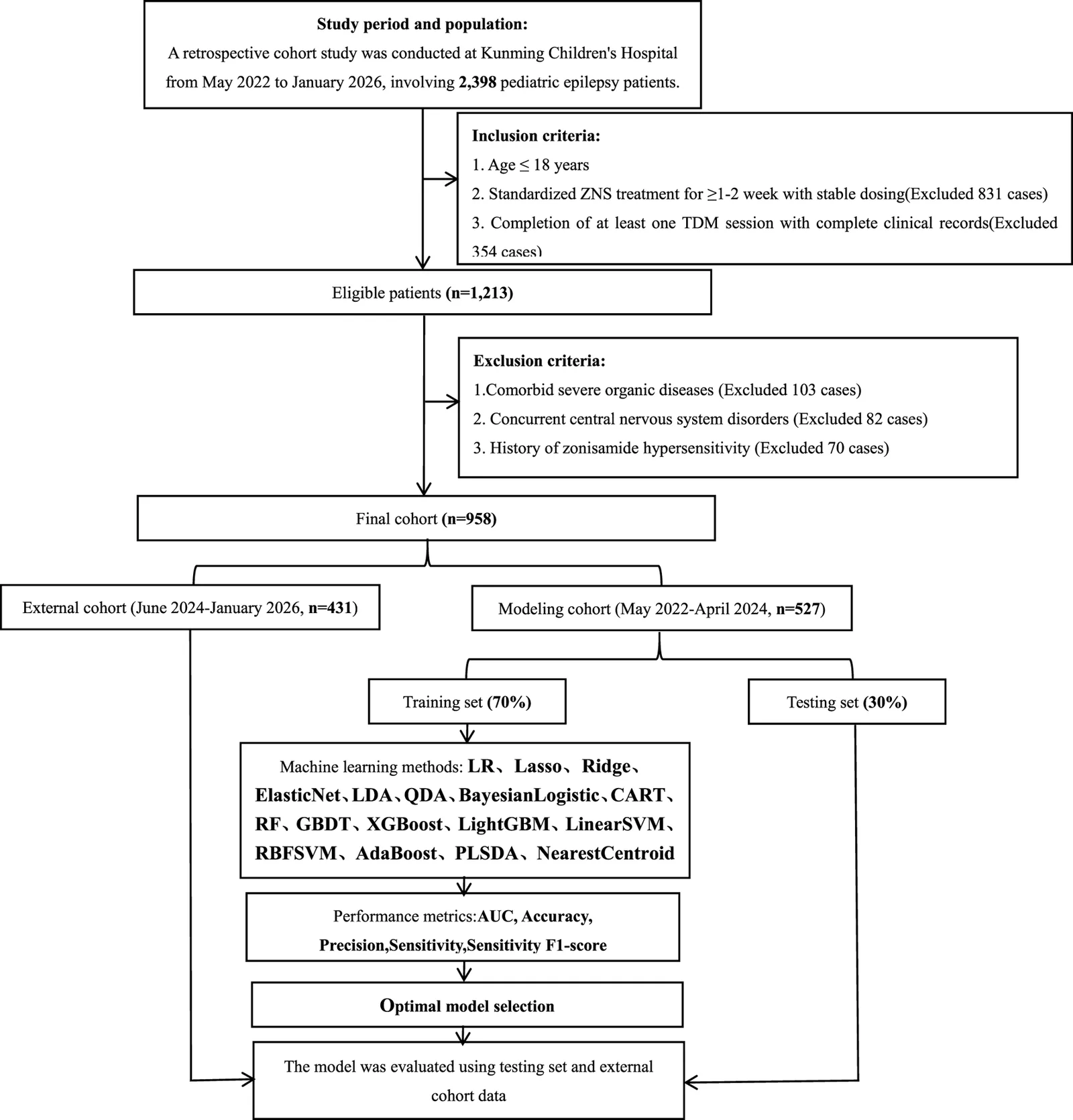
Flowchart of patient screening and machine learning model construction for therapeutic drug monitoring of zonisamide in pediatric patients with epilepsy. Abbreviations: LR, Logistic Regression; Lasso, Least Absolute Shrinkage and Selection Operator; Ridge, Ridge Regression; ElasticNet, Elastic Net; LDA, Linear Discriminant Analysis; QDA, Quadratic Discriminant Analysis; Bayesian Logistic, Bayesian Logistic Regression; CART, Classification And Regression Tree; RF, Random Forest; GBDT, Gradient Boosting Decision Tree; XGBoost, eXtreme Gradient Boosting; LightGBM, Light Gradient-Boosting Machine; LinearSVM, Linear Support Vector Machine; RBFSVM, Radial Basis Function Support Vector Machine; AdaBoost, Adaptive Boosting, PLSDA, Partial Least Squares Discriminant Analysis; Nearest Centroid, Nearest Centroid Classifier; TSIV, Time-Split Internal Validation; AUC, Area Under the Curve.

The modeling cohort of this study included 527 patients with epilepsy who received ZNS treatment at the Department of Neurology, Kunming Children’s Hospital from May 2022 to May 2024, while the time-split internal TSIV cohort comprised 431 patients treated in the same department from June 2024 to January 2026 (the case screening process is shown in Figure 1). In the present study, pediatric patients with epilepsy were defined as children and adolescents aged ≤18 years who sought medical attention for recurrent seizures and received standardized ZNS treatment. The diagnosis was established based on the unified diagnostic criteria for epilepsy outlined in the Clinical Guidelines for Diagnosis and Treatment: Epilepsy (2022 Edition) published by the Editorial Committee of Clinical Diagnosis and Treatment Guidelines of the National Health Commission (Jones et al., 2023). A comprehensive assessment incorporating clinical history, neurological examination, electroencephalography, and neuroimaging was conducted to confirm the diagnosis. Patients with febrile seizures, reflex seizures, or other non-epileptic paroxysmal disorders were excluded. The inclusion criteria were as follows: (1) Diagnosed with epilepsy based on clinical history, neurological examination, and necessary electroencephalography or neuroimaging examinations, excluding febrile seizures, reflex seizures, and other non-epileptic seizure disorders; (2) Age ≤ 18 years (Jones et al., 2023); (3) All samples were collected after the medication regimen had been stabilized and pharmacokinetic steady state had been achieved. In cases where therapeutic parameters, such as dosage adjustments, changes in dosing frequency, or administration timing, were modified, sampling was postponed until a new steady state was re-established. This protocol was implemented to eliminate potential confounding bias arising from fluctuations in drug concentration; (4) Completed at least one ZNS TDM session, possessed complete concentration detection records, and had complete and traceable clinical data including simultaneous liver and kidney function, blood routine examinations, and adverse reaction assessments; (5) Ensured each subject corresponded to only one clinical data record (covering ZNS concentration monitoring data) to achieve data deduplication and avoid duplicate inclusion. The exclusion criteria included: (1) Comorbid severe liver or kidney failure, heart failure, or hematological system diseases; (2) Comorbid other central nervous system diseases (such as encephalitis, meningitis, or brain tumors); (3) Allergy or intolerance to ZNS (graded according to Common Terminology Criteria for Adverse Events version 5.0 (CTCAE 5.0) standards (Frederick and National Cancer Institute, 2017): allergy was defined as the occurrence of drug-specific rash, pruritus, mucosal damage, or systemic hypersensitivity after administration; intolerance referred to the persistence of nervous system, digestive system, or organ-related adverse reactions after administration despite dosage adjustment and symptomatic treatment, rendering standard treatment unsustainable; both were comprehensively determined by specialist physicians based on clinical symptoms, parental feedback, and laboratory examinations); (4) Unauthorized adjustment of ZNS dosage or interruption of treatment without the permission of the attending physician; (5) Patients receiving concomitant medications known to interfere with the metabolism or concentration of ZNS were excluded from the study. Specifically, drugs such as lamotrigine, topiramate, sodium valproate, risperidone, sertraline, and fluoxetine were excluded due to their potential to inhibit hepatic metabolism, thereby elevating ZNS concentrations and increasing the risk of adverse reactions, including sedation and fatigue. Conversely, medications including carbamazepine, phenytoin, phenobarbital, and oxcarbazepine were excluded for their potential to decrease ZNS levels. Furthermore, patients co-administered hepatic enzyme inducers (e.g., rifampicin, glucocorticoids) or inhibitors (e.g., omeprazole, cimetidine, fluconazole) were excluded. These agents may alter cytochrome P450 enzyme activity, thereby disrupting the absorption, metabolism, and excretion of ZNS, compromising concentration stability, and potentially introducing bias into the study results.

### 2.2 Zonisamide dosage regimens

In accordance with the routine clinical protocols of our center and manufacturer guidelines, the ZNS administration regimen was established as follows: the initial dose for pediatric patients was 1.00–2.00 mg/(kg·d), administered orally in one or two divided doses. Dosage adjustments were made every 1–2 weeks based on seizure control and tolerability, with incremental increases not exceeding 2.00 mg/(kg·d). The conventional maintenance dosage ranged from 2.00–4.00 mg/(kg·d); however, for patients with refractory epilepsy or those receiving combination therapy, the dosage could be titrated up to a maximum of 8.00 mg/(kg·d). Appropriate dose increases were required for patients concomitantly receiving hepatic enzyme-inducing anti-seizure drugs. Overall, dosing was individualized based on clinical seizure control, patient tolerability, and TDM results. To ensure comparability, this study utilized the weight-standardized daily dose (mg/(kg·d), calculated as total daily dose divided by measured body weight) as the core variable. Medication was administered orally at fixed times once or twice daily. Samples were collected in the morning under fasting conditions, within 30 min prior to the next scheduled dose, to determine the steady-state trough concentrations of ZNS. This 30-min sampling window was established in accordance with clinical specifications for the TDM of antiseizure medications in children, the pharmacokinetic characteristics of ZNS, and standard protocols for similar TDM studies (Chinese Medical Association, 2023).

This approach balanced the need for analytical accuracy with practical considerations regarding pediatric outpatient workflows and patient compliance. In the modeling cohort, the attainment of pharmacokinetic steady state was verified using measured trough concentration data; this objective confirmation ensured sample baseline homogeneity and the reliability of the subsequent model construction. All enrolled pediatric patients maintained a fixed dosage regimen throughout the study, with no instances of clinical dose adjustments, self-initiated dose modifications, or premature discontinuation. Medication adherence was strictly monitored; the protocol stipulated a missed dose frequency of less than 5% and prohibited consecutive treatment interruptions of 3 days or longer, unauthorized suspensions, or unexplained discontinuation of standard therapy. Single, short-term delays in administration due to minor physical discomfort were permitted, whereas persistent treatment interruptions were strictly prohibited.

### 2.3 Therapeutic drug monitoring methods and grouping

Steady-state trough samples were uniformly collected from all pediatric patients in the morning under fasting conditions: 2.00 mL of peripheral venous plasma was drawn within 30 min prior to the next scheduled dose. Plasma samples were placed in heparin sodium anticoagulant tubes, centrifuged at 3000 ×g for 10 min to separate plasma, and ZNS concentrations were determined using ultra-performance liquid chromatography (UPLC). Samples were pretreated with 7.00% perchloric acid solution, and 20.00 μL of the supernatant was injected for analysis. Chromatographic separation was performed on an ACQUITY UPLC Peptide BEH C18 column (2.10 mm × 50.00 mm, 1.70 μm) with a mobile phase of 0.10% formic acid-acetonitrile (85:15) at a flow rate of 0.20 mL/min. The column temperature was maintained at 40 °C, and detection was performed at a wavelength of 265 nm. The linear range for ZNS concentration was 0.50–50.00 μg/mL (*R* ^2^ > 0.9996). The extraction recovery rate ranged from 96.90% to 103.70%, with intra-day precision < 7.63%, inter-day precision < 8.22%, and stability test RSD < 14.85%.

All ZNS concentrations detected in this study were ≤40.00 μg/mL; no samples exhibited abnormally high concentrations (>40.00 μg/mL). Based on the clinically recommended effective therapeutic concentration range, participants were stratified into two groups: (1) the target attainment group (10.00–40.00 μg/mL) and (2) the sub-therapeutic group (<10.00 μg/mL).

### 2.4 Clinical data collection

Clinical data were retrieved from the hospital’s electronic medical record system and the laboratory information management system. The collected data included the following: (1) Demographic characteristics: age, sex, and body weight; (2) ZNS administration parameters: dosage, duration of therapy, and concomitant medications. In this study, concomitant medications were defined as drugs administered continuously and regularly for a minimum of 7 days during zonisamide treatment. The scope encompassed other anti-seizure drugs, adjunctive therapies for epilepsy, psychotropic agents for psychiatric comorbidities, and drugs modulating hepatic enzymes. The specific classifications of concomitant medications were as follows: other anti-seizure drugs included sodium valproate, levetiracetam, oxcarbazepine, lacosamide, lamotrigine, carbamazepine, and phenobarbital; adjunctive neurological therapies consisted of neurotrophic and brain metabolism-enhancing agents such as mecobalamin, citicoline sodium, and oxiracetam; medications for comorbidities referred to psychotropic drugs used for the long-term management of emotional disturbances and sleep disorders in pediatric patients, including risperidone oral solution, sertraline, fluoxetine, and tiapride; hepatic enzyme inducers included rifampicin and glucocorticoids, whereas hepatic enzyme inhibitors included omeprazole, cimetidine, and fluconazole. Temporary medications administered during hospitalization, short-term intermittent emergency treatments, and transient fluid therapy were excluded from the statistical analysis of concomitant medications; (3) Laboratory findings: white blood cell count (WBC), red blood cell count (RBC), hemoglobin (HGB), hematocrit (HCT), platelet count (PLT), alanine aminotransferase (ALT), aspartate aminotransferase (AST), total bilirubin (TBiL), total protein (TP), albumin (ALB), serum creatinine (SCr), estimated glomerular filtration rate (eGFR), blood urea nitrogen (BUN), uric acid (UA), fasting blood glucose (FBG), and vitamin D (VitD); (4) ZNS concentration measurements.

All clinical and laboratory data were collected after the patients had achieved a stable dosage and pharmacokinetic steady state, and prior to sampling for concentration determination; immediate test results obtained on the day of sampling were excluded. Static demographic variables, such as gender, were derived from baseline data recorded at enrollment, while dynamic clinical and laboratory indicators were based on the most recent measurements obtained prior to steady-state sampling. This data collection protocol ensures the model’s applicability for the prospective prediction of steady-state plasma concentrations and minimizes analytical bias arising from concurrent testing.

### 2.5 Data preprocessing

To ensure the reliability of the results and strictly prevent data leakage, this study implemented a rigorous data preprocessing pipeline for machine learning. All preprocessing parameters were derived exclusively from the training set and subsequently applied to the test and TSIV sets. The specific procedures were as follows: (1) Data partitioning: The modeling cohort was randomly divided into a training set (n = 372) and a test set (n = 159) at a ratio of 7:3. This partitioning standard is widely recognized in clinical predictive modeling and machine learning, balancing the requirements for model training and independent validation. (2) Missing value imputation: Missing values were addressed using random probability imputation based on training set data. Specifically, the distributions of variables were fitted using complete observations from the training set only. For continuous variables, the distribution type was determined first; if a normal distribution was satisfied, random imputation was performed using normal distribution parameters; if normality was achieved following logarithmic transformation, log-normal distribution imputation was applied; and when neither parametric distribution was met, non-parametric sampling based on the empirical cumulative distribution constructed from the training set was used for imputation. For categorical variables, random imputation was performed by fitting a multinomial discrete distribution based on the frequencies in the training set. The distribution models determined by the training set were uniformly applied to impute missing values across the training set, internal test set, and external validation set. The sample sizes and missing rates for each variable are summarized in Supplementary Table S1. (3) Outlier treatment: The interquartile range (IQR) method was employed using training set data to calculate critical values for outlier truncation, thereby preventing data leakage resulting from the incorporation of information from the test or external validation sets into threshold construction. Specifically, the IQR was calculated as Q_3_ (upper quartile) minus Q_1_ (lower quartile) for each continuous variable in the training set. The outlier boundaries were defined as (Q_1_-1.5 × IQR, Q_3_ + 1.5 × IQR). Values falling outside this interval were subjected to winsorization, being replaced by the corresponding interval endpoints. This set of thresholds was uniformly applied to all three datasets—the training set, internal test set, and independent external validation set—without utilizing validation subset data to modify the truncation standards. Statistics regarding IQR-based outlier detection and winsorization for continuous variables across the datasets are presented in Supplementary Table S2. (4) Categorical variable encoding: Dummy coding was performed for binary variables (e.g., gender, combination therapy) based on the distribution in the training set, with consistent encoding rules maintained across all datasets. (5) Continuous variable standardization: All continuous variables were standardized using Z-score normalization based on the mean and standard deviation of the training set, thereby eliminating dimensional differences and ensuring consistency across datasets.

### 2.6 Feature selection

To strictly adhere to the principles of data leakage prevention in machine learning, all feature screening procedures were conducted independently within the training set. The test set and external validation set were excluded from any statistical testing, variable selection, or parameter fitting processes. Feature screening was implemented in the following sequence: (1) Correlation analysis and collinearity diagnosis: First, the Shapiro-Wilk test was employed to assess the normality of continuous variables. Given that the majority of variables in this study did not conform to a normal distribution, Spearman’s rank correlation analysis was uniformly applied to investigate inter-variable associations, thereby circumventing the normality assumption required by Pearson’s correlation analysis. Simultaneously, the Variance Inflation Factor (VIF) was calculated based on the training set data to evaluate multicollinearity. Considering the use of VIF ≥ 10.00 as a sole exclusion criterion to be overly rigid, this study did not directly remove variables with high VIF. Instead, all indicators with clear clinical and biological significance were initially retained; variables exhibiting high VIF but possessing potential clinical value were temporarily preserved to avoid eliminating features of practical research significance based solely on statistical thresholds. Subsequently, Lasso regression and elastic net regularization modeling were employed to further compress redundant collinearity information, balancing statistical collinearity control with the logical rationality of clinical biology. (2) Univariate analysis: Categorical variables were analyzed using the chi-square test, while continuous variables with non-normal distribution were assessed using the Wilcoxon rank-sum test. Variables demonstrating statistical significance in the training set (*P* < 0.05) were retained. (3) Preliminary screening based on Lasso regression: Variables showing statistical significance in the univariate analysis were incorporated into the Lasso regression model. The optimal regularization parameter (λ) was determined via 10-fold cross-validation combined with grid search on the training set. Variables with non-zero regression coefficients and absolute values≥0.05 were retained to eliminate weakly correlated variables, resulting in a candidate feature set. (4) Multi-algorithm joint verification and feature determination: Utilizing the candidate variables retained by Lasso regression, the Boruta algorithm was applied to evaluate feature validity. Only variables with importance significantly higher than shadow features and marked as “Confirmed” were retained. Furthermore, 500-iteration bootstrap resampling were conducted to evaluate screening stability based on variable selection frequency. Ultimately, the core feature set for modeling was established by synthesizing multiple results, including VIF collinearity diagnosis, clinical and biological evidence, Lasso regularization constraints, Boruta feature importance, and Bootstrap screening stability. Once the feature subset was determined, it was directly applied to the test set and the TSIV cohort without conducting secondary screening, threshold adjustment, or feature reconstruction for the external validation cohort. A summary of the original candidate variables, the screened modeling variables, and the theoretical basis for inclusion is provided in Supplementary Table S3.

### 2.7 Construction of multiple machine learning models and selection of the optimal model

This study conducted modeling analysis using R software (version 4.5.2). A random seed of 123 was consistently set to strictly ensure experimental reproducibility. All models shared a unified cross-validation strategy for hyperparameter grid search optimization. A total of 17 binary classification models were constructed for predictive performance comparison, including Logistic Regression (LR), Least Absolute Shrinkage and Selection Operator (Lasso), Ridge Regression (Ridge), Elastic Net (ElasticNet), Linear Discriminant Analysis (LDA), Quadratic Discriminant Analysis (QDA), Bayesian Logistic Regression, Classification and Regression Tree (CART), Random Forest (RF), Gradient Boosting Decision Tree (GBDT), eXtreme Gradient Boosting (XGBoost), Light Gradient-Boosting Machine (LightGBM), Linear Support Vector Machine (LinearSVM), Radial Basis Function Support Vector Machine (RBFSVM), Adaptive Boosting (AdaBoost), Partial Least Squares Discriminant Analysis (PLSDA), and Nearest Centroid classifier.

For linear probability models, LR employed a binomial logit generalized linear model. Lasso, Ridge, and ElasticNet were implemented via the “glmnet” package with alpha fixed at 1.00, 0.00, and 0.50, respectively; the penalty parameter lambda was searched over a grid from 0.05 to 0.50 with a step size of 0.05. Regularization was applied to shrink feature coefficients and suppress model overfitting. Bayesian Logistic Regression was fitted using a Bayesian generalized linear model, relying on prior constraints to further reduce model degrees of freedom. Discriminant analysis models (LDA, QDA, PLSDA) and the Nearest Centroid classifier (based on the pam algorithm) are low-degree-of-freedom linear models inherently free from overfitting risks; PLSDA was tuned with three principal components to reduce interference from redundant features through dimensionality reduction.

For the CART, the rpart algorithm was employed with the minimum split sample size (minsplit) set to 15.00. The complexity parameter (cp) was optimized within the range of 0.01–0.10 with a step size of 0.01. Cost-complexity pruning was performed based on cross-validation error. Tree growth was strictly limited by dual constraints on the minimum node sample size and the complexity parameter, thereby structurally preventing overfitting, stabilizing model output, and enhancing reproducibility.

Ensemble tree models uniformly employed shallow weak learners to reduce model capacity. Random Forest was configured with 30.00 decision trees, a maximum node count (maxnodes) of 3.00 per tree, and a minimum terminal node size (nodesize) of 15.00; the number of features sampled (mtry) was optimized by traversing values up to one-third of the total features, leveraging random sampling for inherent noise reduction. GBDT and AdaBoost both utilized decision trees with a depth of 1.00, a learning rate of 0.10, and a minimum node sample size of 15.00, with iteration counts of 30.00 and 50.00, respectively. XGBoost utilized a binary logistic loss objective, with a maximum tree depth of 1.00, a learning rate of 0.10, and 30.00 iterations. LightGBM employed binary logistic loss, limiting the maximum tree depth to 1.00 and the number of leaves per tree to 4.00, with 30 iterations. The combination of shallow trees and limited iteration rounds effectively prevented overfitting to training set noise. For Support Vector Machine models (LinearSVM and RBFSVM), the penalty parameter C was optimized via grid search within the range of 0.01–0.20; RBFSVM fixed the kernel coefficient sigma at 0.10, balancing fitting bias and variance through the penalty term.

Multiple structural constraints, regularization strategies, and a standardized tuning process collectively ensured that all models were free from overfitting. The entire modeling workflow utilized fixed parameters and a unified random seed, ensuring robust reproducibility. Upon completion of training, all models output positive prediction probabilities for each sample for subsequent performance evaluation.

All models were constructed based on the feature variables obtained via uniform screening. Hyperparameter optimization was performed within the training set using grid search combined with 10-fold cross-validation to determine the optimal model architecture and parameter combinations for each algorithm. Model performance was comprehensively evaluated with the Area Under the Receiver Operating Characteristic Curve (AUC) as the core metric, incorporating a multi-dimensional comparison of model fitting and generalization capabilities. By comparing the predictive efficacy across the training and validation sets of 17 candidate models, the model demonstrating the highest comprehensive performance and generalization stability was selected as the final prediction model.

Subsequent robustness verification and in-depth analysis—including discrimination validation, calibration assessment, Decision Curve Analysis (DCA) for clinical benefit, robustness testing via 500-bootstrap resampling, SHAPley Additive exPlanations (SHAP) interpretability analysis, and feature decision logic mining—were exclusively conducted on the selected optimal model. This focused approach eliminated redundant validation of alternative models, thereby streamlining the research scope to highlight the clinical application value and intrinsic decision mechanisms of the optimal model.

### 2.8 Statistical analysis and model evaluation

All statistical analyses were performed using R software (version 4.5.2). Continuous variables with a non-normal distribution were expressed as median (interquartile range) [M (Q1, Q3)], and between-group comparisons were conducted using the Mann-Whitney U test. Categorical variables were presented as frequencies (percentages) [n (%)], with group differences assessed using the chi-square (χ^2^) test. A two-sided P-value < 0.05 was considered statistically significant.

Model performance was comprehensively evaluated across four dimensions: discrimination, calibration, clinical applicability, and basic metrics, in both the modeling/testing cohort and the external validation cohort. Based on the binary classification outcomes, the confusion matrix parameters were defined as follows: True Positive (TPos) referred to samples with a positive actual outcome correctly predicted as positive; False Positive (FPos) referred to samples with a negative actual outcome incorrectly predicted as positive; True Negative (TNeg) referred to samples with a negative actual outcome correctly predicted as negative; and False Negative (FNeg) referred to samples with a positive actual outcome incorrectly predicted as negative. The calculation formulas for the evaluation metrics were as follows:

Accuracy reflected the overall correctness of the model predictions:

$$\text{Accuracy}=\left(\text{TP}+\text{TN}\right)\;/\;\left(\text{TP}+\text{FN}+\text{FP}+\text{TN}\right).$$

Precision measured the proportion of true positives among all samples predicted as positive, emphasizing the control of false positive misjudgments:

$$\text{Precision}=\text{TP }/\;\left(\text{TP}+\text{FP}\right).$$

Sensitivity reflected the model’s ability to identify positive cases, focusing on minimizing false negative omissions:

$$\text{Sensitivity}=\text{TP }/\;\left(\text{TP}+\text{FN}\right).$$

Specificity measured the ability of the model to correctly identify true negative cases, representing the proportion of actual negative samples accurately predicted as negative:

$$\text{Specificity}=\text{TN}/\left(\text{TN}+\text{FP}\right).$$

The F1-score, calculated as the harmonic mean of Precision and Sensitivity, was used to comprehensively balance the predictive efficacy of the model:

$$F1=2\times \text{Precision}\times \text{Sensitivity}/\left(\text{Precision}+\text{Sensitivity}\right).$$

Model discrimination was evaluated using the Receiver Operating Characteristic (ROC) curve and the AUC. The AUC and its 95.00% confidence interval were calculated using 500-iteration bootstrap resampling. Calibration was assessed using calibration curves, Brier scores, slope, and intercept to evaluate the consistency between predicted probabilities and actual event rates; calibration curves were fitted using 500-iteration bootstrap resampling. Clinical utility was assessed using DCA to quantify the net benefit across different threshold probabilities, comparing the model against the strategies of “intervening in all patients” and “intervening in none.”

## 3 Results

### 3.1 Baseline characteristics

A total of 958 pediatric patients were enrolled in this study and divided into a modeling cohort (n = 527) and a TSIV cohort (n = 431). In the modeling cohort, there were 308 males (58.44%) and 219 females (41.56%), with a median age of 8.67 (6.92, 10.75) years (range: 1.08–17.60 years) and a median weight of 31.00 (22.60, 40.60) kg (range: 8.33–73.10 kg). The target ZNS concentration was achieved in 259 patients (49.15%). In the TSIV cohort, there were 249 males (57.77%) and 182 females (42.23%), with a median age of 9.73 (6.88, 11.67) years (range: 1.42–15.69 years) and a median weight of 31.05 (22.65, 40.50) kg (range: 9.43–66.63 kg). The target ZNS concentration was achieved in 259 patients (60.09%). There were no statistically significant differences in baseline characteristics between the two groups (*P* > 0.05), indicating comparability (Table 1).

**TABLE 1:** Comparison of baseline characteristics in children.

Variable	Modeling cohort (n = 527)	TSIV cohort (n = 431)	*P* value
Gender (male/female)	308/219	249/182	0.89
Concomitant (0/1)	431/96	359/72	0.60
Weight (kg)	31.00 (22.60,40.60)	31.05 (22.65,40.50)	0.894-
Age (years)	8.67 (6.92,10.75)	9.73 (6.88,11.67)	0.77
Dose (mg/(kg・d))	2.42 (2.10,2.69)	2.43 (2.12,2.69)	0.75
Duration (Months)	9.30 (4.96,15.75)	9.30 (4.90,15.75)	0.95
ALT (U/L)	16.00 (13.00,20.00)	16.00 (13.00,20.00)	0.87
AST (U/L)	24.00 (20.00,28.00)	24.00 (20.00,29.00)	0.77
TBIL (μmol/L)	10.90 (8.80,13.500)	10.90 (8.60,13.50)	0.84
TP (g/L)	72.90 (68.60,76.22)	72.90 (68.25,76.10)	0.76
ALB (g/L)	43.30 (41.30,45.70)	43.20 (41.50,45.65)	0.97
UA (μmol/L)	288.70 (254.15,355.25)	287.90 (251.80,346.05)	0.75
BUN(mmol/L)	4.89 (4.16,5.69)	4.88 (4.16,5.58)	0.93
SCr(μmol/L)	47.00 (39.00,56)	47.00 (39.00,56.00)	0.72
GFR (mL/(min·1.73 m^2^))	100.39 (83.56,129.57)	101.13 (84.35,129.57)	0.71
FBG (mmol/L)	5.26 (4.88,5.60)	5.26 (4.89,5.60)	0.90
WBC(10^9^/L)	6.43 (5.58,7.62)	6.43 (5.62,7.56)	0.95
RBC(10^12^/L)	4.93 (4.62,5.20)	4.94 (4.62,5.19)	0.96
HGB (g/L)	138.00 (133.00,145.50)	138.00 (133.00,145.00)	0.84
HCT (10^9^/L)	40.50 (38.60,42.60)	40.60 (38.50,42.60)	0.92
PLT (%)	298.00 (262.50,357.00)	295.00 (255.00,358.00)	0.81
VitD (nmol/L)	62.41 (51.62,72.00)	62.13 (46.91,71.93)	0.34

Continuous variables are expressed as median (interquartile range, IQR), while categorical variables are expressed as counts (n). Comparisons between groups were performed using the Mann-Whitney U test (for continuous variables) and the χ^2^ test (for categorical variables), with *P* < 0.05 considered statistically significant. Abbreviations: ALT, alanine aminotransferase; AST, aspartate aminotransferase; TBIL, total bilirubin; TP, total protein; ALB, albumin; UA, uric acid; BUN, blood urea nitrogen; SCr, serum creatinine; GFR, glomerular filtration rate; FBG, fasting blood glucose; WBC, white blood cell; RBC, red blood cell; HGB, hemoglobin; HCT, hematocrit; PLT, platelet; Vit, D: vitamin D; TSIV, Time-Split Internal Validation.

### 3.2 Screening of key influencing factor

#### 3.2.1 Correlation analysis and multicollinearity diagnosis

**FIGURE 2:**
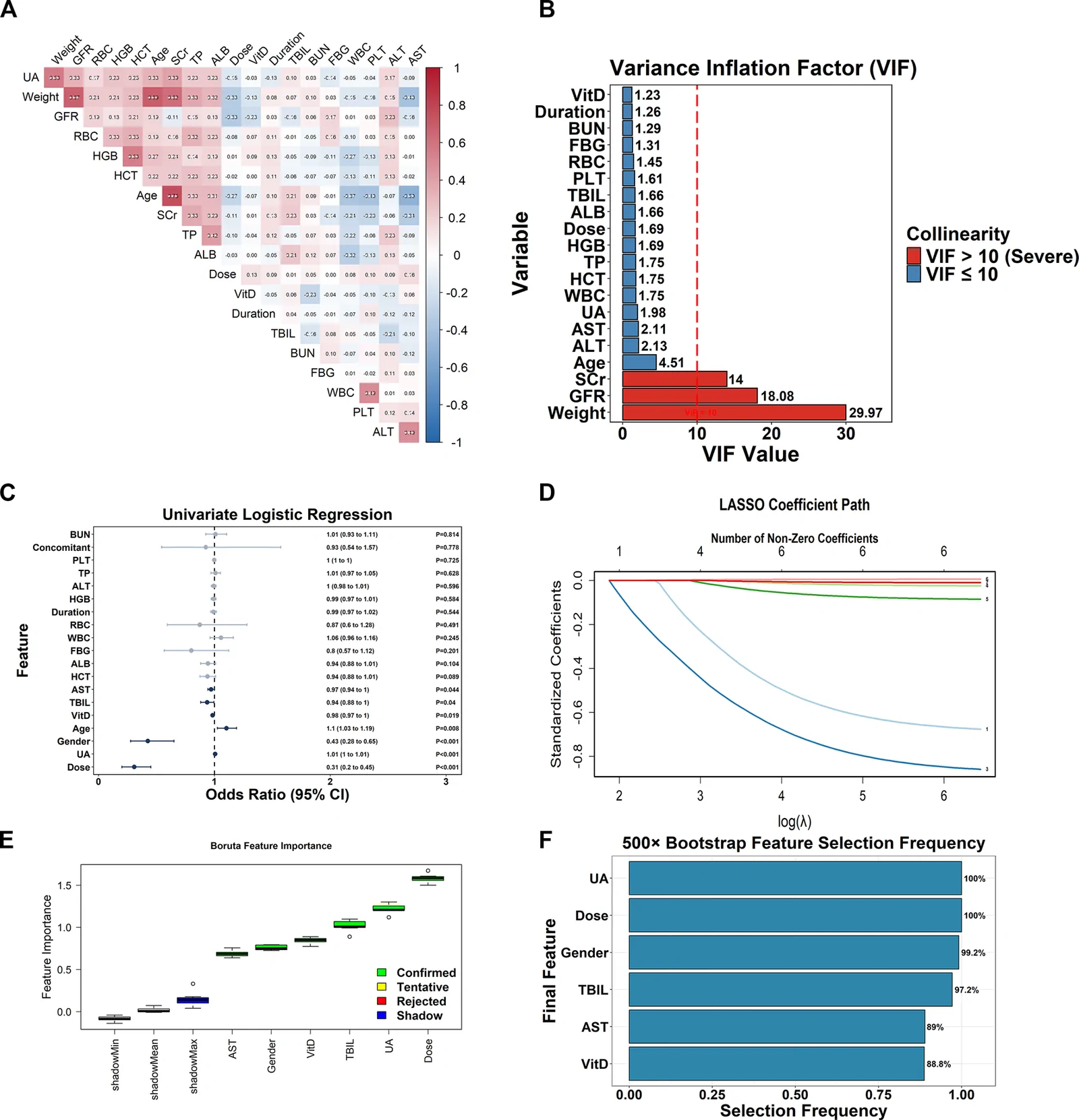
Screening results for key factors influencing subtherapeutic zonisamide concentrations in pediatric patients with epilepsy. **(A)** Heatmap of the correlation matrix for clinical characteristic variables. The heatmap displays pairwise Spearman correlation coefficients among demographic, clinical, and laboratory parameters. Red indicates a positive correlation, while blue indicates a negative correlation; color intensity is proportional to the magnitude of the correlation coefficient (range: −1.00 to 1.00). **(B)** Variance Inflation Factor (VIF) of clinical characteristics used to assess multicollinearity. The red dashed line represents the critical threshold (VIF = 10.00); variables with VIF > 10.00 suggest significant multicollinearity. **(C)** Forest plot of univariate linear regression for clinical characteristic variables in the training set. The plot illustrates the regression coefficients (β-coefficient) and their 95% confidence intervals (95% CI) for each variable. Blue dots represent the estimated regression coefficients, and horizontal lines represent the 95% CI. Statistical significance in univariate analysis (*P* < 0.05) is indicated when the confidence interval does not cross the null line (β = 0.00, represented by the red dashed line). **(D)** Trajectory of feature coefficients versus -log(λ) (the negative logarithm of the regularization parameter λ) in Lasso regression. As -log(λ) increases (indicating weakened regularization intensity), the regression coefficients of features gradually deviate from zero and converge to their final model values. The vertical axis (Coefficients) represents the regression coefficient values with zero as the baseline; the horizontal axis (-log(λ)) represents the logarithmic transformation of regularization strength, where larger values indicate weaker regularization constraints. **(E)** Box plot of feature importance for each variable under 10-fold cross-validation of the Random Forest model, illustrating the distribution of contribution across different data subsets. The vertical axis represents the feature importance score, and the horizontal axis lists variable names. The width and position of the boxes reflect the stability and relative magnitude of feature importance; green boxes represent variables with high stability and contribution. **(F)** Bar chart of feature selection frequency for each variable under 500-iteration bootstrap resampling. The vertical axis lists variable names, and the horizontal axis represents the selection frequency during bootstrap resampling (range: 0.00–1.00); higher frequencies suggest stronger predictive stability of the variable. Abbreviations: ALT, Alanine aminotransferase; AST, Aspartate aminotransferase; TBIL, Total bilirubin; TP, Total protein; ALB, Albumin; UA, Uric acid; BUN, Blood urea nitrogen; SCr, Serum creatinine; GFR, Glomerular filtration rate; FBG, Fasting blood glucose; WBC, White blood cell; RBC, Red blood cell; HGB, Hemoglobin; HCT, Hematocrit; PLT, Platelet; Vit. D: Vitamin D.

Spearman correlation analysis was employed to evaluate the associations between subtherapeutic ZNS concentrations and clinical variables in pediatric patients with epilepsy (Figure 2A). The results indicated a moderate positive correlation between body weight and age (r = 0.69). In contrast, correlations between liver and kidney function markers (including TBIL, TP, and UA) as well as Vitamin D and other variables were weak, with correlation coefficients consistently below 0.30. Multicollinearity diagnostics (Figure 2B) revealed VIFs ranging from 1.23 to 29.37. Notably, body weight (VIF = 29.37), GFR (VIF = 18.98), and serum creatinine (SCr, VIF = 6.51) exhibited VIFs exceeding 10, indicating significant multicollinearity. From a physiological perspective, the concurrent increase in body weight with age in children constitutes a natural physiological coupling, which predisposes the model to multicollinearity bias. Furthermore, the physiological metabolic interdependence among GFR, SCr, and body weight further exacerbates this collinearity interference.

#### 3.2.2 Univariate analysis of factors influencing ZNS concentration

Univariate analysis revealed that dosage, UA, gender, age, weight, vitamin D, TBIL, and AST were significantly associated with subtherapeutic ZNS concentrations in pediatric patients with epilepsy (*P* < 0.05); conversely, the remaining variables showed no significant association (*P* > 0.05) (Figure 2C). Considering both statistical collinearity and pediatric physiological characteristics, a strong correlation was observed between weight and age. Simultaneous inclusion of these variables could lead to biased model parameter estimation. To mitigate the interference of multicollinearity in subsequent regression modeling, and based on clinical context and the collinearity criterion of a VIF≥10, the variable “weight” was excluded from further analysis.

#### 3.2.3 Lasso regression analysis

A Lasso regression model was applied to further screen the seven characteristic variables that showed statistical significance in the univariate analysis. The optimal regularization parameter (λ) was determined via grid search combined with 10-fold cross-validation. The coefficient path plot illustrated that as the value of −log(λ) increased (i.e., λ decreased), the regression coefficients of the variables gradually deviated from zero. At the optimal λ value (0.04), the coefficient for age, was compressed to zero, suggesting no significant association with subtherapeutic ZNS concentrations. The remaining variables were retained in the model, identifying them as potential influencing factors (Figure 2D).

#### 3.2.4 RF feature importance ranking

To enhance the stability and rationality of feature selection, the Boruta algorithm was utilized to evaluate feature importance, using the candidate variables retained by the Lasso regression as inputs. The results showed that the importance of all included candidate variables (dose, UA, TBIL, VitD, gender, and AST) was significantly higher than the maximum value of the shadow features, and all were marked as “confirmed.” This indicates that these variables made substantial contributions to the predictive performance of the model. Importance ranking revealed that dosage had the highest importance score, followed by UA; both were significantly higher than other variables. Although gender and AST showed relatively lower importance, they remained significantly higher than the shadow feature levels and were therefore retained (Figure 2E).

#### 3.2.5 Stability assessment via 500-iteration bootstrap resampling

To further verify the stability of the feature selection, 500-iteration bootstrap resampling were performed to evaluate the inclusion frequency of candidate variables (Figure 2F). The results indicated that the inclusion frequencies for the final selected variables—dose, UA, TBIL, VitD, gender, and AST-were all above 85.00%, suggesting robust stability in the feature selection results. Based on a multidimensional assessment integrating clinical physiological evidence, collinearity diagnostics, univariate analysis, Lasso regularization screening, Boruta feature importance ranking, and Bootstrap stability validation, six key variables were ultimately identified as predictors of concentration attainment status for the subsequent construction of the ZNS binary classification prediction model.

### 3.3 Performance comparison of machine learning models

**FIGURE 3:**
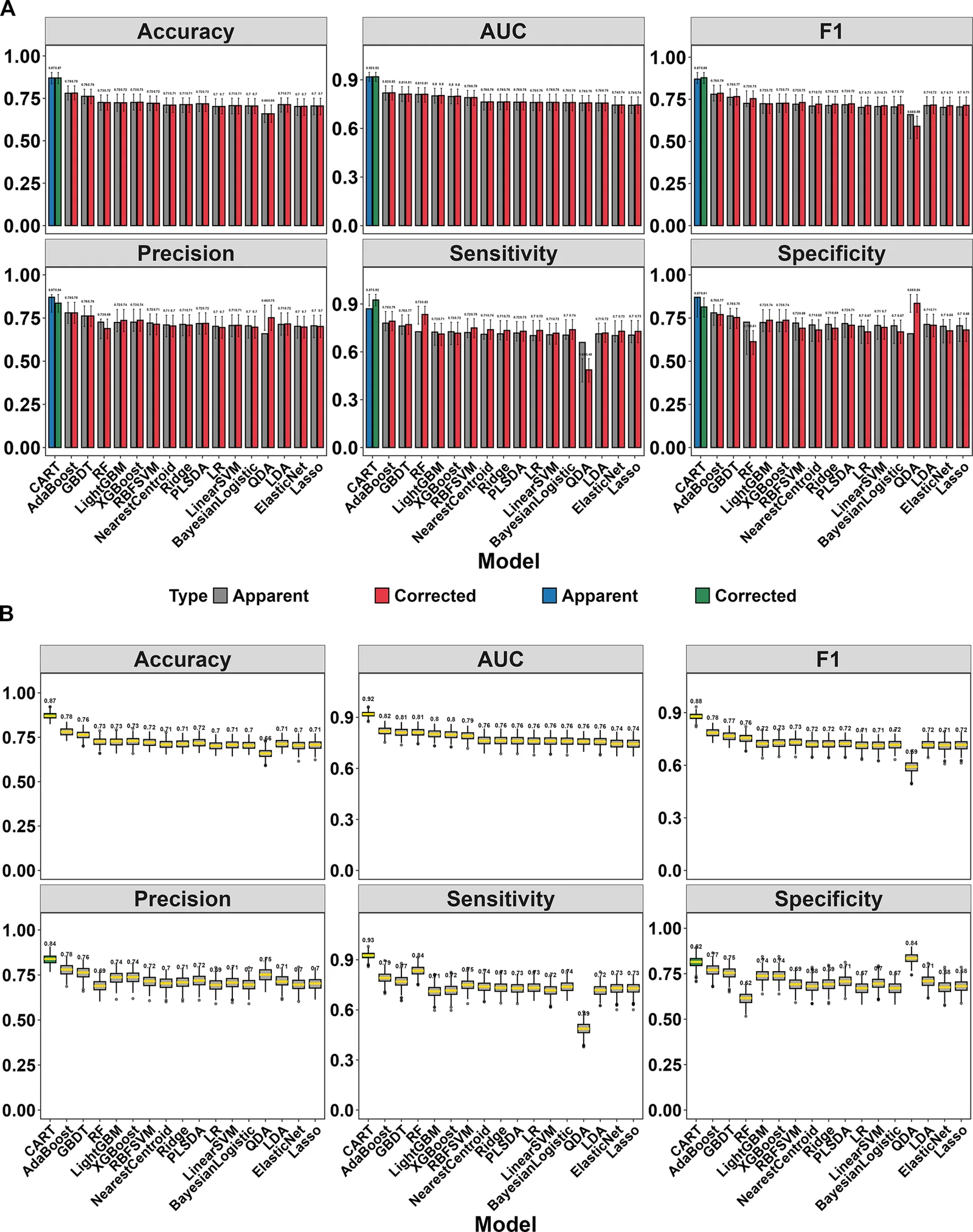
Performance evaluation of the models. **(A)** Comparison of predictive performance for various regression models before and after calibration. The figure illustrates the variations in metrics—including Accuracy, AUC, F1-score, Precision, Sensitivity, and Specificity—across 17 models evaluated on the training set and via internal validation using 1,000-iteration bootstrap resampling. **(B)** Box plots depicting the distribution of metrics derived from the 1,000 bootstrap resampling iterations, visually reflecting the fluctuation characteristics and stability differences in the predictive performance of the distinct models. Model abbreviations: LR, Logistic Regression; Lasso, Least Absolute Shrinkage and Selection Operator; Ridge, Ridge Regression; ElasticNet, Elastic Net; LDA, Linear Discriminant Analysis; QDA, Quadratic Discriminant Analysis; Bayesian Logistic, Bayesian Logistic Regression; CART, Classification And Regression Tree; RF, Random Forest; GBDT, Gradient Boosting Decision Tree; XGBoost, eXtreme Gradient Boosting; LightGBM, Light Gradient-Boosting Machine; LinearSVM, Linear Support Vector Machine; RBFSVM, Radial Basis Function Support Vector Machine; AdaBoost, Adaptive Boosting; PLSDA, Partial Least Squares Discriminant Analysis; Nearest Centroid, Nearest Centroid Classifier.

This study compared the predictive efficacy of 17 classification models using the training set, with all evaluation metrics undergoing bias correction via 1,000 -iteration bootstrap resampling. The bar chart (Figure 3A) reveals a distinct stratification in the predictive capabilities of the models. The CART model achieved the highest performance across key metrics, both before and after correction, with an Accuracy of 87.00%, AUC of 91.80%, F1-score of 87.80%, Precision of 83.60%, Sensitivity of 92.50%, and Specificity of 81.40%, significantly outperforming all other models. Ensemble models, including AdaBoost, GBDT, RF, LightGBM, and XGBoost, ranked closely behind, demonstrating comprehensive efficacy second only to CART.

The box plots of bootstrap resampling distributions (Figure 3B) further validated the performance stability of the models. The CART model exhibited compact box distributions with low dispersion and prominent median values across all evaluation metrics, showing minimal overall fluctuation. This indicates high consistency in prediction results during repeated resampling and superior model robustness. Conversely, some traditional statistical models (e.g., Bayesian Logistic, QDA) displayed wide box spans and dispersed metric distributions accompanied by significant result fluctuations, suggesting unstable predictive performance and weak generalization capabilities. Collectively, the visualization results demonstrate that the CART model possesses significant advantages in prediction accuracy, bias control, and stability upon repeated validation, establishing it as the optimal prediction model in this study.

### 3.4 Robustness assessment of the optimal model

#### 3.4.1 Discrimination assessment

The prediction model was constructed using the CART algorithm, which was identified as having the optimal comprehensive performance. Discrimination was assessed via 500-iteration bootstrap resampling to verify the robustness of the model’s discriminative efficacy across the training set, the internal test set, and the TSIV set. The ROC curve results for each cohort are presented in the corresponding figures.

**FIGURE 4:**
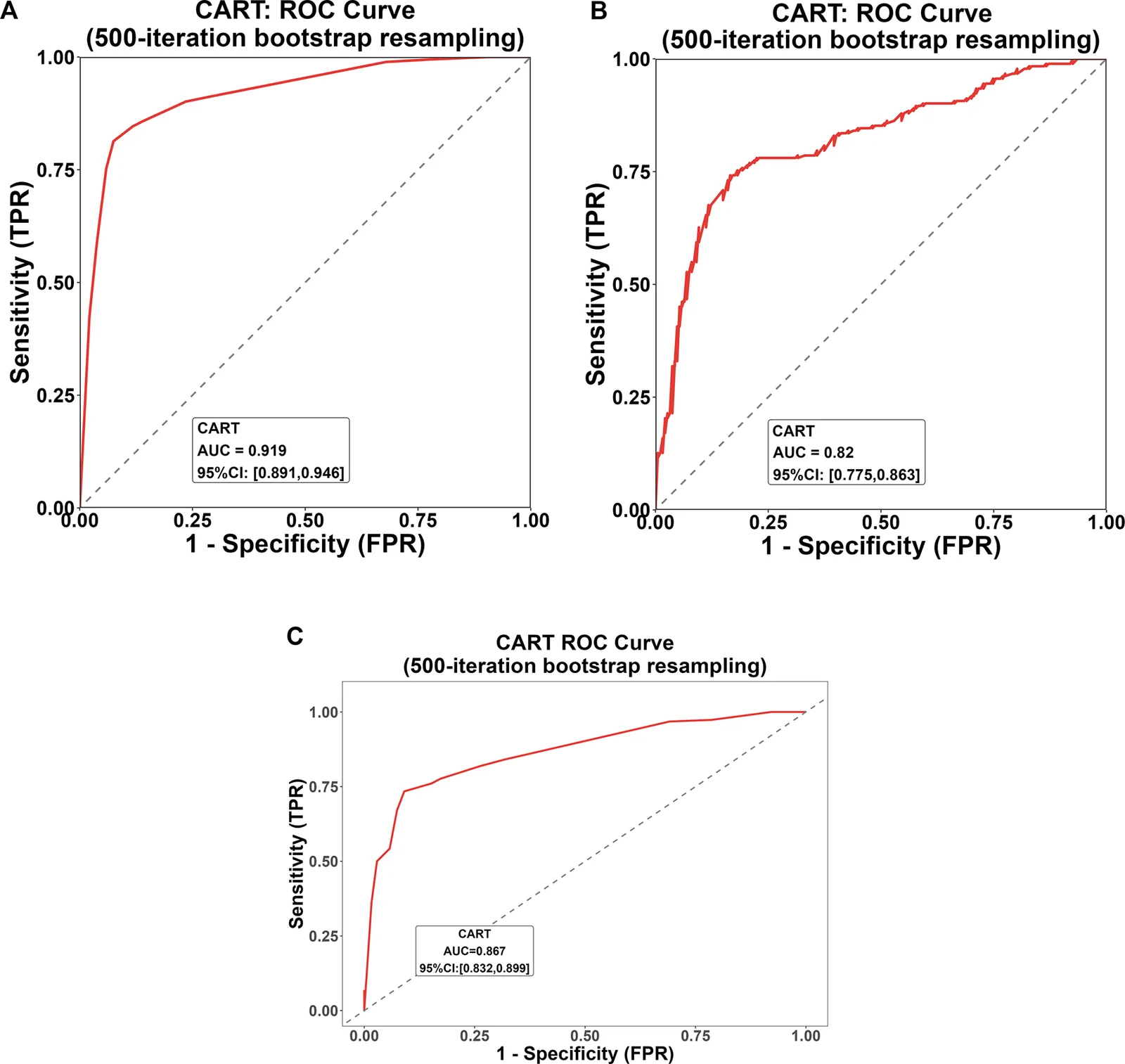
ROC curves for the binary classification CART model predicting ZNS concentration in children with epilepsy. The figure illustrates the ROC curves for the optimal CART model across the training, testing, and external validation sets. Model performance is indicated by the proximity of the curve to the upper left corner. **(A)** ROC curve for the training set (AUC = 0.92, 95% CI: 0.89–0.95). **(B)** ROC curve for the testing set (AUC = 0.82, 95% CI: 0.78–0.86). **(C)** ROC curve for the TSIV set (AUC = 0.87, 95% CI: 0.83–0.90).

In the training set (Figure 4A), the model achieved a post-calibration AUC of 0.92 (95% CI: 0.89–0.95). According to the grading standards for clinical prediction model efficacy, an AUC value≥0.90 indicates that the model demonstrates excellent discriminative performance in the training cohort, effectively distinguishing between target events and non-event samples. The ROC curve was positioned close to the top-left corner, indicating no significant deviation.

In the internal test set (Figure 4B), the model yielded an AUC of 0.82 (95% CI: 0.78–0.86), falling within the 0.75–0.90 range, which suggests that the model maintained good discriminative efficacy during internal validation. The difference in AUC between the training and test sets was merely 0.099, indicating no significant performance degradation. This suggests the absence of severe overfitting and confirms excellent stability in internal validation.

In the TSIV set (Figure 4C), the model achieved an AUC of 0.87 (95% CI: 0.83–0.90). As this value is ≥ 0.85, the model demonstrates superior discriminative ability in the TSIV cohort. The performance attenuation in TSIV compared to the training set was minimal, and the 95% CI was narrow, indicating stable generalization performance and strong reproducibility across temporal scenarios.

Collectively, the validation results across the three cohorts demonstrate that the AUC values of the CART model in the training, internal test, and TSIV sets were significantly higher than the random level (AUC = 0.70). Furthermore, the absence of significant performance decay in TSIV further confirms the model’s robust discriminative ability and cross-cohort generalization potential, providing a reliable predictive basis for clinical decision-making.

#### 3.4.2 Calibration curve assessment

**FIGURE 5:**
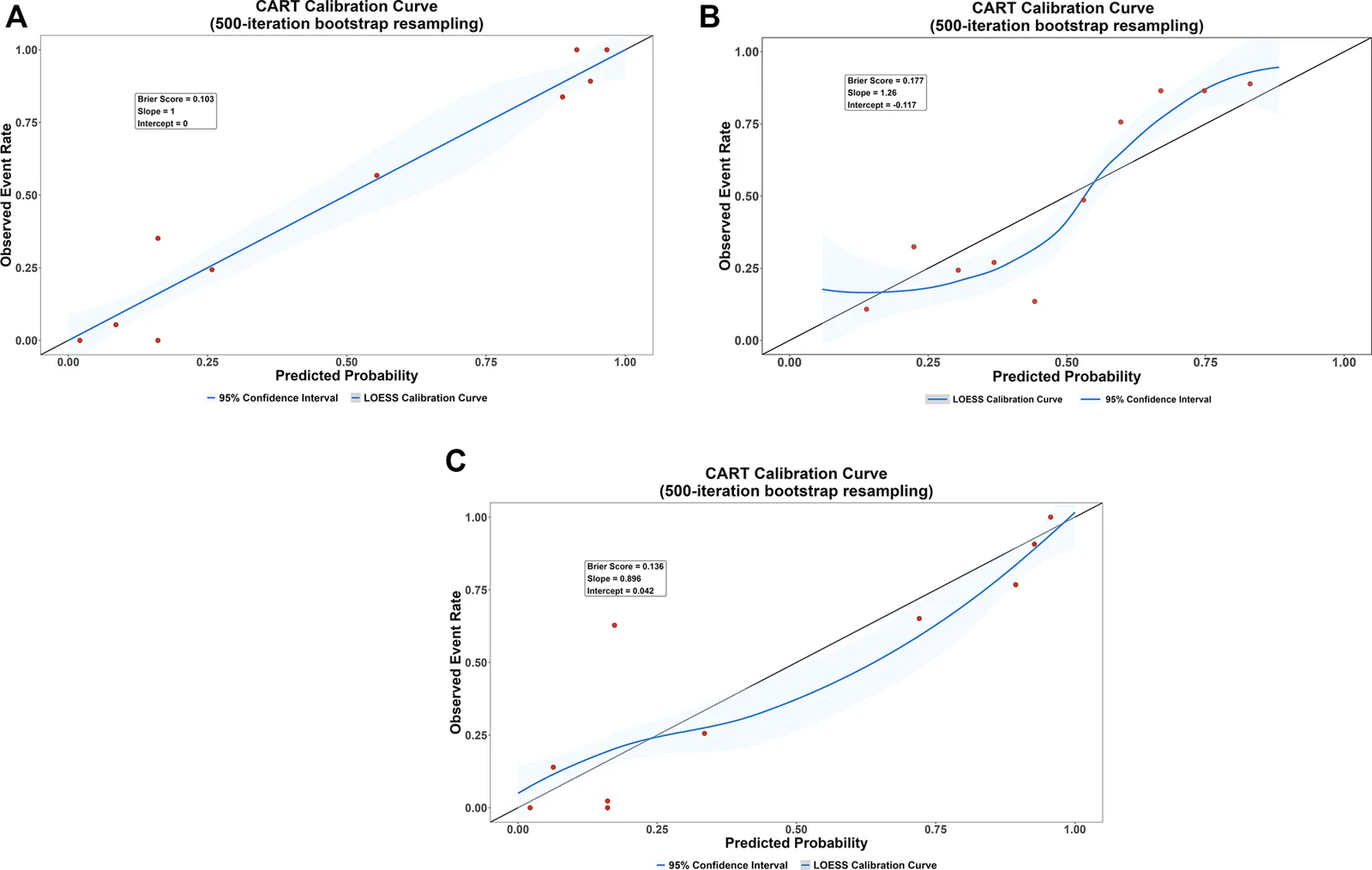
Calibration curves for the binary classification prediction of ZNS concentration in children with epilepsy using the CART model. Note: Calibration curves of the prediction model across different cohorts. Bias-corrected calibration was assessed using 500-iteration bootstrap resampling. **(A)** Calibration curve for the training set. The bias-corrected curve closely aligned with the ideal 45-degree diagonal line. The Brier score was 0.10, with a calibration slope of 1.00 and an intercept of 0.00, indicating a high consistency between the predicted probabilities and the actual observed event rates. **(B)** Calibration curve for the testing set. The Brier score was 0.18, with a calibration slope of 1.26 and an intercept of −0.12. The curve closely approximated the diagonal line, and the 95% confidence interval fully encompassed the diagonal, indicating no significant deviation. **(C)** Calibration curve for the TSIV set. The Brier score was 0.14, with a calibration slope of 0.90 and an intercept of 0.04. The curve generally aligned with the diagonal, showing only minor deviations in the medium-to-high probability range, which demonstrates good calibration performance and stable generalizability of the model.

A prediction model was constructed utilizing the optimal CART algorithm selected through comprehensive screening. Calibration was evaluated using 500-iteration bootstrap resampling to rigorously assess model performance across the training, internal testing, and TSIV sets; the calibration curves for each cohort are presented in Figure 5.

In the training set (Figure 5A), the calibration curve, corrected via 500-iteration bootstrap resampling, demonstrated high concordance with the ideal diagonal line. The Brier score was 0.10, with a calibration slope of 1.00 and an intercept of 0.00, indicating high consistency between predicted probabilities and observed event rates without systematic bias. In the testing set (Figure 5B), the Brier score was 0.18. The calibration curve closely approximated the diagonal line, with a slope of 1.26 and an intercept of −0.12. Although slight underestimation was observed in the low-risk range and minor overestimation in the high-risk range, the 95% confidence interval encompassed the diagonal line throughout, indicating no significant deviation. In the TSIV set (Figure 5C), the Brier score was 0.14, with a slope of 0.90 and an intercept of 0.04. The overall trend of the curve closely aligned with the diagonal, with only minor deviations in the medium-to-high probability range, suggesting that the model maintained acceptable calibration performance in the TSIV cohort.

Overall, the Brier scores exhibited a gradual increase across the training, internal testing, and TSIV sets, consistent with the natural attenuation pattern of model generalization, and no severe calibration failure was observed.

#### 3.4.3 Clinical applicability

**FIGURE 6:**
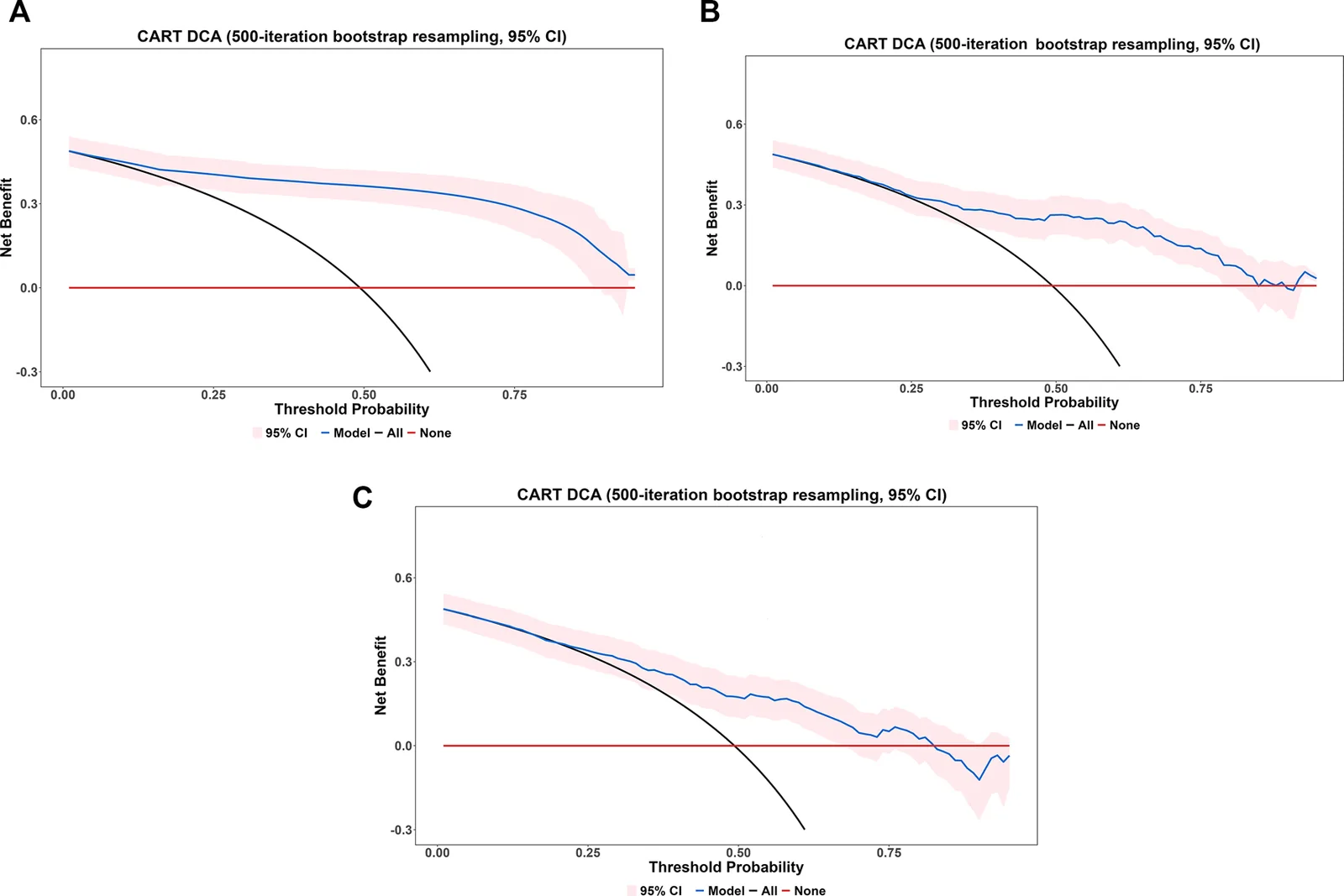
Decision curve analysis for the validation of the CART prediction model for ZNS concentration in children with epilepsy. Clinical net benefits of the CART prediction model were evaluated using 500-iteration bootstrap resampling with 95% confidence intervals (CI). **(A)** Training cohort; **(B)** Internal validation cohort; **(C)** TSIV cohort. The blue curves represent the net benefit of the model, while the black curves and red horizontal lines represent the “all intervention” and “no intervention” strategies, respectively. The model curves demonstrated significantly higher net benefits than both reference lines across all cohorts. Furthermore, stable net benefits were observed within a broad threshold probability range (0.00–0.80), indicating substantial clinical utility and application value for the prediction model.

DCA was employed to evaluate the clinical utility of the CART prediction model (Figure 6). In the training set (Figure 6A), the net benefit curve of the model was significantly higher than the “treat-all” and “treat-none” reference lines within a threshold probability range of 0.00–0.80. This suggests that utilizing the model for intervention guidance yields a stable net benefit across a broad spectrum of clinical decision thresholds. Similarly, in the internal testing set (Figure 6B) and the TSIV set (Figure 6C), the net benefit curves remained significantly superior to the reference lines within the 0.00–0.80 threshold probability range. Furthermore, the 95% confidence intervals did not intersect with the reference lines, indicating that the model exhibits robust and stable clinical decision-making efficacy in both internal and TSIV cohorts, thereby demonstrating high potential for clinical application.

#### 3.4.4 Feature importance and decision logic analysis of the CART model

**FIGURE 7:**
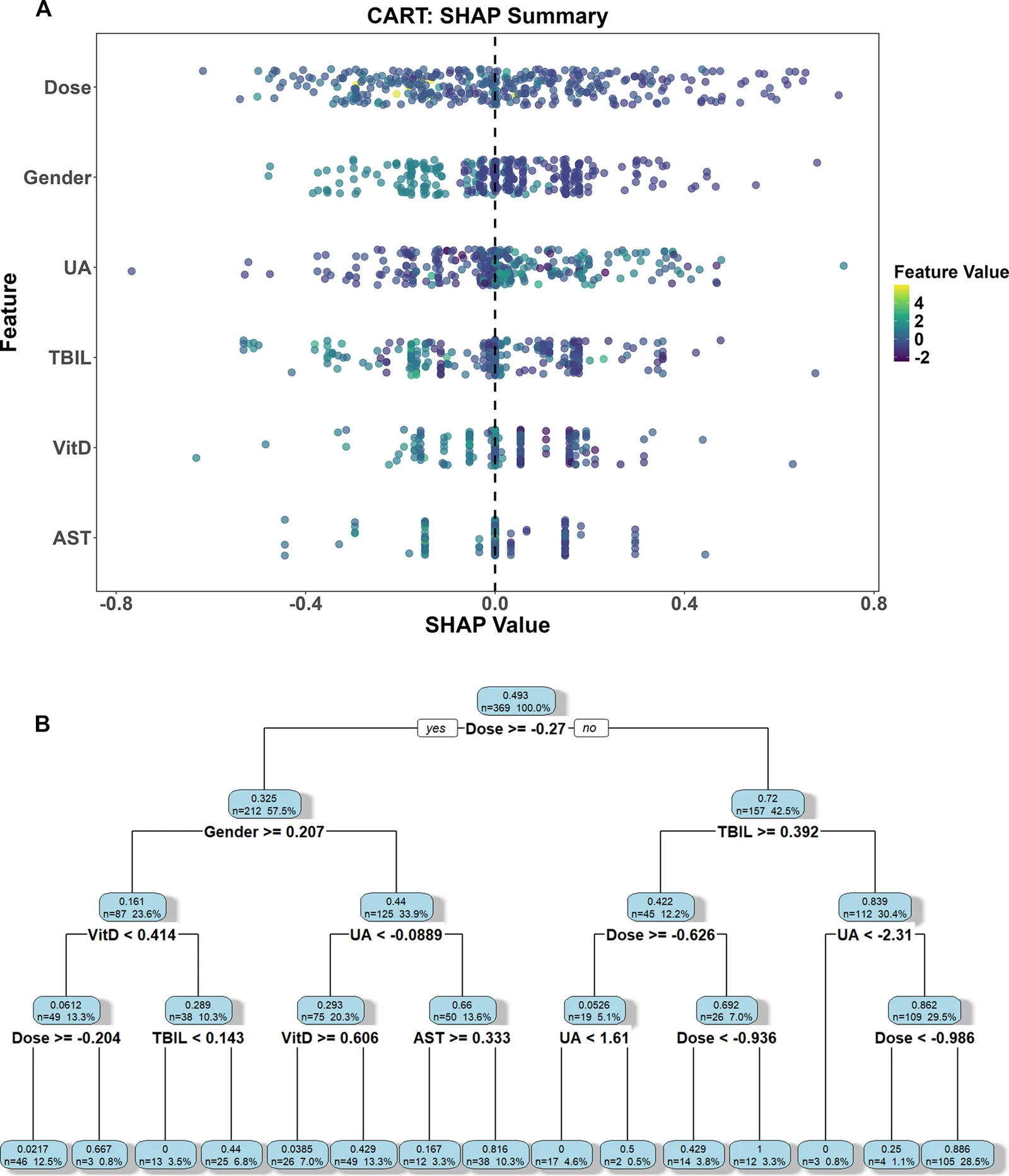
Multidimensional interpretability analysis of the CART algorithm model. **(A)** SHAP feature contribution summary plot. The vertical axis ranks features in descending order of importance, while the horizontal axis represents the SHAP value. The color of the points indicates the magnitude of the feature value, and their distribution reveals the direction and strength of the influence of feature values on the prediction results. **(B)** Representative decision tree of the CART algorithm model (4-layer depth, training set). The values within the nodes represent the average predicted drug concentration for that branch, with the sample size and proportion annotated below. This visualization intuitively demonstrates the hierarchical interactive decision rules of the features.

To further elucidate the intrinsic decision-making mechanism of the CART algorithm model, this study employed the SHAP method for global interpretation and utilized a representative decision tree to visually present feature interaction rules. The results are as follows:

The SHAP summary plot (Figure 7A) illustrates the ranking of feature importance: Dose, Gender, UA, TBIL, VitD, and AST were identified as key factors influencing the discrimination of concentration target achievement status. Among these, Dose and Gender exhibited the widest range of SHAP values, indicating the greatest magnitude of impact on the discrimination results of target achievement outcomes. Dose demonstrated an overall contribution trend: high-dose samples (yellow/green points in the figure) corresponded to positive SHAP values, suggesting that higher dosages increase the probability of achieving the target concentration; conversely, low-dose samples exhibited negative contributions, reducing the likelihood of target achievement, which aligns with basic pharmacokinetic principles. As a binary feature, the SHAP values for Gender presented a distinct bimodal distribution, indicating systematic differences between sexes regarding the probability of target achievement, with specific gender subgroups significantly elevating or reducing the risk of failing to achieve the target drug concentration. Regarding laboratory indicators such as UA, TBIL, VitD, and AST, the distribution of SHAP values revealed nonlinear associations with target achievement status. For instance, samples with higher UA levels mostly corresponded to negative SHAP values, suggesting that high UA is associated with an increased risk of subtherapeutic drug concentrations; TBIL, VitD, and AST exhibited more complex bidirectional effects, where their levels could alter the probability of target achievement in different directions. The SHAP value distributions of these features were relatively concentrated, contributing less to the discrimination results than Dose and Gender, yet they remained important modulating factors in the model’s decision-making.

The representative decision tree of the CART model (Figure 7B) visually demonstrates the hierarchical interaction logic of features. At the root node (first layer), the model first utilized Dose (Dose ≥ −0.27) as the primary splitting feature to divide samples into two main branches, directly reflecting the dominant influence of dosage on whether the target concentration was achieved, consistent with the importance ranking in the SHAP analysis. At the second layer, following dose stratification, the model employed Gender (Gender≥0.21) and Total Bilirubin (TBIL ≥ 0.39) for secondary splitting in the two branches, respectively, to further refine risk subgroups. This suggests that gender and liver function indicators are key factors modulating the probability of target achievement after dose stratification. For example, in the high-dose branch, Gender divided samples into subgroups with different baseline risks for target achievement; in the low-dose branch, TBIL level became the key stratifying factor, suggesting that liver function status has a more prominent impact on the probability of target achievement in the low-dose population. At the third and fourth layers, the model introduced indicators such as VitD, UA, AST, and TBIL for finer partitioning, forming multiple terminal risk nodes. For instance, in the high-dose female subgroup, VitD levels provided further stratification, where low VitD combined with high TBIL further reduced the probability of target achievement; in the low-dose high-TBIL subgroup, UA level interacted with dosage to jointly determine the final discrimination result, with low UA combined with lower dosage significantly increasing the risk of subtherapeutic concentration.

The decision tree clearly demonstrates the hierarchical decision logic of the model: using dosage as the core driving factor and combining it with the interactions of multiple indicators—including gender, liver function (TBIL, AST), renal function (UA), and nutritional status (VitD)—the model establishes multidimensional discrimination rules for the risk of zonisamide concentration target achievement, corroborating the feature importance and contribution patterns revealed by the SHAP analysis. Synthesizing the SHAP analysis and decision tree results, the CART binary classification model constructed in this study possesses interpretable decision logic consistent with pharmacokinetics and clinical knowledge: the administered dose is the core variable regulating the probability of target concentration achievement, while gender, indicators reflecting liver metabolism and protein binding function (TBIL, AST), renal excretion-related metabolic indicators (UA), and nutritional status markers (VitD) jointly participate in regulating *in vivo* drug exposure levels. This fully reflects the characteristic that the entire process of drug absorption, distribution, metabolism, and excretion is influenced by multiple physiological factors. The aforementioned results confirm that the stratification rules used by the CART model to discriminate concentration target achievement are highly consistent with existing pharmacokinetic theories, providing a sufficient interpretable basis for the model’s clinical application in rapidly screening pediatric patients at high risk of subtherapeutic concentrations.

## 4 Discussion

Pediatric patients with epilepsy exhibit significant heterogeneity in physiological metabolism. Variations in liver and kidney function, developmental status, nutritional status, and endocrine function directly influence the absorption, metabolism, and clearance of anti-seizure medications. These factors are primary contributors to fluctuations in ZNS concentrations and unstable therapeutic efficacy. As a first-line broad-spectrum ASM, ZNS is widely used in both monotherapy and adjunctive therapy for pediatric epilepsy. Maintaining steady-state trough concentrations within an appropriate therapeutic window is crucial for ensuring seizure control and minimizing the risk of adverse reactions (Mandrioli et al., 2021; Fu et al., 2025). Compared to adults, pediatric patients demonstrate greater inter-individual variability in ZNS concentrations and are subject to more complex influencing factors. Consequently, traditional empirical dose adjustment models are often inadequate for meeting the requirements of individualized precision therapy. Therefore, the integration of multidimensional clinical indicators to conduct intelligent prediction of concentration target achievement and analysis of risk factors holds significant clinical relevance and scientific value.

Current research on the mechanisms and concentrations of ZNS predominantly relies on classical population compartmental pharmacokinetic models, with conclusions largely derived from adult cohorts. Large-scale, real-world, multi-indicator integrated prediction studies focusing on pediatric populations remain scarce. In this study, utilizing a large-scale retrospective cohort of pediatric patients with epilepsy, we successfully constructed a prediction model for the attainment status of ZNS concentrations. This was achieved through rigorous standardized variable screening, parallel comparison of multiple machine learning algorithms, and multi-stage internal validation alongside time-series external validation. Machine learning and classical compartmental pharmacokinetic models possess distinct theoretical advantages and application scenarios; they do not exhibit a mutually exclusive relationship but rather complement each other, synergistically serving the goals of precision medicine research.

Current research on the mechanism and concentration of ZNS predominantly relies on classical population compartmental pharmacokinetic models, with conclusions largely derived from adult cohorts. Consequently, large-sample, real-world, multi-indicator integrated studies on the classification prediction of concentration attainment in pediatric populations remain scarce. Based on a large retrospective cohort of pediatric patients with epilepsy, this study successfully constructed a binary classification prediction model for the target attainment status of ZNS steady-state trough concentrations. This was achieved through rigorously standardized variable screening, parallel comparison of multiple machine learning algorithms, and both internal validation and TSIV. Machine learning and classical compartmental pharmacokinetic models possess distinct theoretical advantages and application scenarios. They do not exhibit a mutually exclusive relationship; rather, each has unique strengths, mutually supporting one another to collaboratively advance research on precision medication.

### 4.1 Analysis of key factors influencing ZNS concentrations in pediatric patients with epilepsy

Throughout this study, strict data leakage prevention strategies were implemented. All variable screening, multicollinearity testing, univariate analysis, and feature optimization were conducted exclusively within the training set. Through a comprehensive multi-dimensional analytical framework encompassing clinical physiological mechanisms, multicollinearity diagnosis, univariate analysis, lasso regression screening, boruta feature importance ranking, and bootstrap stability verification, highly collinear indicators such as body weight were excluded. Ultimately, dosage, age, sex, UA, TBIL, AST, and VitD were identified as core influencing factors. This approach effectively mitigated variable redundancy and collinearity bias, ensuring the stability and reliability of the risk factor screening results.

The administered dosage directly reflects the intensity of individualized dosing. Both excessive and insufficient dosages can compromise the attainment of target concentrations, rendering dosage a critical indicator for concentration regulation. Kimura et al. (Kimura et al., 1992) found a significant positive correlation between weight-adjusted daily dose and steady-state concentration, and Silva et al. (Silva et al., 2025) similarly observed that ZNS trough concentrations increased with dosage. These findings align with the results of the present study, confirming that both absolute dose and weight-adjusted dose significantly influence ZNS concentration. However, significant inter-individual variability exists, and dose adjustment is not a simple linear calculation. A study involving European adults with refractory epilepsy indicated that despite the use of high-dose ZNS (median dose 633 mg/d), two patients with measurable levels remained within the therapeutic range, suggesting that even at higher doses, concentrations did not exceed the therapeutic window (Miro, et al., 2016). Therefore, in clinical practice, reliance solely on fixed doses or linear extrapolation should be avoided in favor of multi-factor integrated individualized dosing strategies.

Current clinical pharmacokinetic data regarding ZNS in patients with renal impairment remain limited. Zonisamide is primarily excreted via the kidneys as the parent drug and metabolites, with the clearance process involving both tubular secretion and glomerular filtration; consequently, renal function status directly modulates systemic drug exposure (Miura, 2004). Immature renal development or abnormalities in renal function indices (such as uric acid metabolic disorders) can reduce the zonisamide clearance rate via mechanisms including competition for tubular excretion and decreased glomerular filtration efficiency, thereby potentially inducing fluctuations in plasma drug concentrations (Anderson and Hakimian, 2014). Pediatric patients, characterized by immature renal metabolic function and limited regulatory capacity for antiepileptic drug excretion, are particularly susceptible to interference from biochemical abnormalities, which increases the risk of plasma drug concentrations falling outside the therapeutic range. Therefore, close therapeutic drug monitoring and renal safety assessment are warranted when administering ZNS to patients with renal impairment.

Gender reflects the physiological disparities in the growth and development of children. A population pharmacokinetic study by Qiu et al. (2016) involving Chinese healthy volunteers demonstrated significant sex-based differences in ZNS concentrations, indicating that sex influences inter-compartmental clearance. Conversely, Silva et al. (2025) concluded that sex does not affect ZNS clearance. This discrepancy may stem from differences in study populations: Silva et al. focused on adult patients, whereas the present study centered on pediatric patients. Physiological differences between sexes during childhood—such as body weight, fat distribution, and the maturation of hepatic and renal function—are more pronounced, potentially leading to sex-dependent disparities in drug metabolism and distribution. Leppik (2004) reported that while ZNS exhibits low binding affinity to plasma proteins, its affinity for RBC is eightfold higher, resulting in significant drug accumulation within human RBC. Given that males typically possess higher RBC counts than females, this may result in stronger drug-RBC binding in male patients, followed by slower drug release and elimination. This mechanism may elucidate the lower peak concentrations and slower elimination rates observed in male patients, highlighting the distinct influence of sex on the pharmacokinetics of zonisamide in children. A single-dose study in Chinese healthy volunteers also revealed sex-based differences in Cmax; however, an analysis of pediatric patients showed no significant difference in clearance between males and females (Dai, 2014). These variations may be attributed to the regulation of drug-metabolizing enzymes by sex hormones, sex-based differences in volume of distribution caused by body fat proportions, and variations in gastrointestinal absorption rates (Smith et al., 2022). Although these differences may attenuate after achieving steady-state with long-term administration, sex remains a factor warranting consideration during the initial treatment phase or dose adjustment.

Levels of TBIL and AST serve as indicators of hepatic metabolic and protein-binding functions. Impaired hepatic function may compromise ZNS clearance and protein binding, resulting in abnormal drug concentrations (Huang et al., 2021). A reported case of ZNS-associated acute-on-chronic liver failure manifested as jaundice and mild encephalopathy after 13 days of administration, with laboratory findings indicating significant hepatic dysfunction (103-fold increase in ALT, 44-fold increase in AST, and 10-fold increase in TBIL) (Kimura et al., 1992). This evidence suggests that patients with hepatic insufficiency may exhibit impaired ZNS metabolism, necessitating close monitoring of liver function and drug concentrations. The findings of the present study support this perspective, emphasizing the importance of ZNS concentration monitoring and safety assessment for patients with hepatic or renal insufficiency.

This study is the first to incorporate VitD levels into a prediction model for achieving target ZNS concentrations in pediatric patients, confirming that low VitD levels constitute an independent risk factor for failure to reach the therapeutic threshold. Bilge and Taşkın (2025) further corroborated the association between VitD status and the stability of ZNS concentrations, suggesting its utility as an adjunctive indicator for therapeutic drug monitoring. These findings indicate that clinical dosing regimens should account for VitD levels to facilitate individualized dosing and improve therapeutic target attainment rates.

Therefore, to optimize the therapeutic efficacy and safety of ZNS, a “multifactorial dynamic assessment” strategy is recommended. During the initial treatment phase, baseline factors such as age and sex should be considered. Throughout the course of treatment, regular monitoring of hepatic and renal function is essential; dosing regimens should be dynamically adjusted by integrating therapeutic drug monitoring results with patient compliance.

### 4.2 Performance comparison of machine learning models and selection of the optimal model

Classical compartmental pharmacokinetic models are underpinned by clear physiological mechanisms and rigorous mathematical frameworks, offering distinct advantages in dynamic quantitative analysis. By relying on fixed compartment structures and kinetic equations, these models can accurately simulate the time-course of drug *in vivo* extending beyond steady-state trough concentration predictions to include precise estimations of drug concentrations at arbitrary time points, the area under the AUC, clearance, volume of distribution, and time to reach steady state. Consequently, they can comprehensively characterize the entire process of drug exposure, making them suitable for mechanistic elucidation, dynamic dose titration, and standardized pharmacokinetic research, serving as a cornerstone for individualized drug dosing (Okada et al., 2008).

Concurrently, machine learning algorithms offer significant application advantages over traditional pharmacokinetic models. Compartmental models rely heavily on pre-defined equations and pharmacokinetic data, limiting their efficacy in analyzing complex multi-indicator interactions, nonlinear associations, and the superimposed effects of confounding variables typical of real-world clinical settings. In contrast, machine learning does not require the prior specification of variable relationships; instead, it autonomously identifies latent associations among routine clinical examinations, demographic characteristics, and biochemical indicators to achieve binary classification of concentration attainment. This approach is particularly well-suited to real-world research scenarios involving pediatric populations, which are characterized by high heterogeneity and multiple confounding factors (Hashimoto et al., 1994; Zhang et al., 2026). Furthermore, regarding the integration of routinely available clinical indicators, large-sample risk stratification, and rapid visual discrimination, machine learning offers operational simplicity, strong scalability, and a lower implementation threshold, thereby better aligning with the requirements of primary clinical care and routine TDM.

This study included 17 mainstream machine learning classification models, employing a unified methodology of 10-fold cross-validation, grid search hyperparameter optimization, and high-order bootstrap bias correction to quantitatively compare discrimination, specificity, sensitivity, and overall stability. The results indicated that traditional linear models had limited fitting efficacy, while ensemble tree models exhibited some fluctuation due to overfitting. The CART model demonstrated the best comprehensive performance due to its structural simplicity, strong interpretability, intuitive hierarchical logic, and stability during repeated validation. This finding aligns with existing research on anti-epileptic drugs; studies on carbamazepine, phenobarbital, phenytoin, and valproic acid have shown that the predictive performance of artificial intelligence models generally surpasses that of traditional population pharmacokinetic models, with ensemble learning methods (e.g., Adaptive Boosting, Extreme Gradient Boosting, and Random Forest) performing particularly well (Chung and Lee, 2025). However, these prior studies did not involve classification prediction. In this study, a LightGBM model within an ensemble learning framework was used to construct a binary classification model for ZNS concentration, which demonstrated superior predictive performance, generalization ability, and clinical applicability compared to similar ensemble models.

These results highlight the diversity and scenario-specific adaptability of machine learning models. Compared to complex “black-box” algorithms, lightweight decision tree models are more easily understood and implemented in clinical settings. Compared to compartmental models with fixed formulas, ML is more adept at extracting real-world patterns from massive clinical datasets. Thus, the two technical systems complement each other (Bamgboye et al., 2026). Their core advantages can be synergistically applied to advance precision medication for pediatric anti-epileptic therapy. On one hand, compartmental pharmacokinetic models focus on mechanism and quantification, capable of dynamically simulating drug concentration changes across different doses and time periods, and precisely calculating core parameters such as AUC, clearance, and peak time. They remain the core means for in-depth mechanistic research, precise dose adjustment, and standardized pharmacokinetic pattern exploration. On the other hand, machine learning possesses strong data mining and clinical translation capabilities, efficiently screening potential confounding factors and identifying nonlinear interactions. It can provide preliminary data support and variable references for compartmental pharmacokinetic research by efficiently screening potential covariates, simplifying model indicators, and optimizing the fitting efficiency of population pharmacokinetic models. In clinical practice, a complementary model can be established: utilizing machine learning models for rapid large-scale preliminary screening and early warning of high-risk populations within the hospital to improve TDM efficiency, followed by refined quantitative assessment and individualized dose titration using compartmental pharmacokinetic models for high-risk pediatric patients. This facilitates a stratified management mode of “broad coverage screening + precision quantification” (Cui et al., 2025; Chusiri et al., 2026).

### 4.3 Robustness, interpretability, and clinical utility of the CART model

This study established a three-tier evaluation framework comprising a training set, an internal test set, and a TSIV set, combined with 500-iteration bootstrap resampling to comprehensively verify model stability. The CART model maintained robust discriminatory performance across multiple cohorts, with calibration curves demonstrating strong agreement with actual clinical event distributions and reasonable fluctuations in Brier scores. DCA confirmed that the model provides stable net benefits across a wide range of clinical thresholds, fully demonstrating the model’s generalizability and clinical applicability (Vickers and Elkin, 2006; Efthimiou et al., 2024).

This study established a three-tier evaluation system comprising a training set, an internal test set, and a TSIV set, combined with 500-iteration bootstrap resampling to comprehensively verify model stability. The CART model, utilized to discriminate whether ZNS concentrations reached the target level, maintained robust discriminatory performance across multiple cohorts. The calibration curve closely aligned with the actual distribution of clinical events, Brier score fluctuations remained within a reasonable range, and DCA confirmed that the model provided stable net benefits across a wide range of clinical thresholds. These findings indicate that the model possesses robust internal discriminatory performance within the population of this center (Vickers and Elkin, 2006; Efthimiou et al., 2024).

By leveraging SHAP interpretability analysis and structured decision tree visualization, this study addressed the “black box” limitation inherent in traditional machine learning. It clearly elucidated the model’s hierarchical discriminative logic, which centers on dosage while integrating hepatorenal function, nutritional indicators, and demographic characteristics. These findings are highly consistent with the physiological mechanisms of zonisamide metabolism (Chen et al., 2026).

From a clinical application perspective, this machine learning model enables rapid assessment utilizing routine admission laboratory indicators, obviating the need for complex pharmacokinetic testing or cumbersome formula calculations. It facilitates large-scale screening for the risk of subtherapeutic concentrations and the early identification of high-risk individuals, thereby significantly enhancing the targeting and efficiency of TDM. Consequently, this approach provides a lightweight and readily deployable intelligent assessment tool for clinical practice, exemplifying the unique core value of machine learning in the domain of precision medicine (Sayadi et al., 2026).

Compared with previous studies (Anderson and Hakimian, 2014), this research exhibits several distinct features: (1) This study utilized a large-sample, real-world retrospective cohort from a single center, incorporating a TSIV set. Despite the balanced baseline and the lack of direct evidence for extrapolation to pediatric populations in other regions, the results confirmed the good internal reproducibility of the machine learning-based target achievement prediction model within the local study population. (2) Data leakage was strictly prevented throughout the process, with standardized variable screening and model training workflows, ensuring methodological rigor; (3) The combination of multi-algorithm comparisons, multiple robustness checks, and interpretability analysis renders the conclusions objective and reliable; (4) Focusing on the special population of children with epilepsy, this study addresses a research gap by enabling intelligent prediction of multi-dimensional influencing factors and target achievement risks associated with ZNS.

This study has several limitations. First, the data were derived exclusively from a single center involving pediatric patients with epilepsy. Consequently, factors such as dietary management, health education, TDM follow-up standards, and combination medication interventions exhibited center-specific characteristics. The temporal stability TSIV only verified the internal reproducibility of the model within the local population and cannot demonstrate its generalizability to pediatric patients across different regions and healthcare systems. Future studies require multi-center, multi-regional prospective independent external cohort validation to confirm the model’s extrapolative value. A critical limitation is that the modeling process employed strict screening criteria for concomitant medications, excluding a large number of patients receiving other antiseizure medications or multiple adjunctive therapies. Polytherapy can alter ZNS metabolic clearance through multiple pathways, including cytochrome enzyme induction/inhibition, transporter competition, and plasma protein displacement, representing the most common and significant confounding factor in clinical practice. However, constrained by the study design, the model lacks data support for patients on polytherapy, which directly limits its generalizability in routine real-world clinical settings. As most pediatric patients with refractory epilepsy are treated with two or more antiseizure medications, this model is applicable only to those on ZNS monotherapy or minimal low-interference adjunctive therapy. It cannot be directly extended to patients on polytherapy, resulting in a narrow scope of clinical application. This represents the primary shortcoming for clinical translation. Future cohorts should expand inclusion criteria to incorporate subgroups of various combination therapies for stratified modeling to enhance predictive capability in polytherapy scenariosy (Collins et al., 2024). Second, due to the retrospective nature of medical records, this study could not include core potential predictors such as drug metabolism gene polymorphisms, standardized medication adherence scores, precise epilepsy syndrome classification, detailed seizure classification, and complete lists of concomitant ntiseizure medications and adjunctive medications. Gene polymorphisms in the CYP and SLC families of drug transporters and metabolic enzymes are innate core drivers of individual pharmacokinetic heterogeneity. Fluctuations in medication adherence directly alter baseline steady-state trough concentrations. Different epilepsy syndromes and seizure types indirectly regulate drug distribution and clearance via neuroendocrine pathways. Furthermore, polytherapy involves significant enzymatic interactions that substantially alter ZNS metabolism rates. These missing variables can independently or interactively affect plasma drug concentrations. Future prospective studies should refine case collection dimensions and expand feature sets to further improve model prediction accuracy (Mgidal et al., 2024). Third, the constructed model can only qualitatively distinguish between two outcomes—target attainment versus non-attainment of ZNS concentration-and cannot precisely predict the actual plasma ZNS concentration values. Future research could integrate regression-based machine learning algorithms and population compartment pharmacokinetic models for joint modeling to simultaneously achieve stratified screening of high-risk populations and precise quantitative calculation of concentrations. Fourth, the core clinical value of TDM ultimately lies in clinical hard endpoints such as seizure control, maintenance of long-term seizure freedom, incidence of adverse drug reactions, treatment tolerance, and drug withdrawal/switching events. However, this study only modeled the intermediate biomarker (plasma drug concentration) without conducting inter-group comparisons of seizure control, adverse events, and treatment termination rates across different concentration strata. Consequently, it cannot directly confirm that optimizing concentration target attainment rates via the model improves long-term epilepsy prognosis. Constrained by retrospective data, follow-up duration varied significantly among patients, standards for recording seizure grading were inconsistent, and underreporting of mild, occult adverse reactions was common, making standardized outcome association analysis difficult after adjusting for confounding factors. Future prospective cohorts will uniformly standardize records of seizure frequency, adverse reaction grading, and treatment duration to fully elucidate the evidence chain of “model-predicted high risk→individualized dose adjustment→improved concentration target attainment rate→improved clinical epilepsy outcomes.” Fifth, this study adopted a fixed therapeutic window of 10.00–40.00 μg/mL for ZNS plasma concentration as the criterion for target attainment, which presents significant methodological and clinical limitations. This general population reference interval may not accommodate individualized differences in pediatric epilepsy patients. Influenced by developmental fluctuations in age, weight, hepatic and renal function, and plasma protein levels, the metabolism, distribution, and free drug activity of ZNS in children exhibit distinct individual differences. A fixed threshold may induce concentration determination bias, causing systematic error in the binary classification outcome of the model (Wallander et al., 2014; Nersesjan et al., 2025). Furthermore, the fixed therapeutic window does not account for the prevalent polytherapy scenarios in real-world clinical practice. Concomitant antiseizure medications can alter ZNS metabolic rates through mechanisms such as hepatic enzyme regulation and transporter competition; thus, a single standard cannot adapt to polytherapy populations, further exacerbating the limited applicability caused by the exclusion of combination therapy cases (Sills and Brodie, 2007). Additionally, this fixed interval was not individually corrected based on key variables such as drug metabolism gene polymorphisms, epilepsy classification, and medication adherence, ignoring pharmacokinetic and disease phenotype heterogeneity. Future prospective multi-center studies are needed to construct a pediatric-specific stratified and individually corrected therapeutic window, integrating physiological characteristics, combination medications, genetic features, and clinical outcomes.

In summary, based on a large-sample real-world retrospective cohort of pediatric epilepsy, this study employed strict data leakage prevention, multi-dimensional feature screening, and multi-level validation to identify core physiological, biochemical, and demographic indicators affecting ZNS plasma concentration in children. Through performance comparison of multiple machine learning algorithms, a CART binary classification model for concentration target attainment was established, demonstrating optimal comprehensive efficiency and good interpretability. This model can rapidly identify pediatric patients with suboptimal ZNS plasma concentrations using routine clinical tests, serving as a preliminary screening tool for patients under the same diagnostic and treatment model in the hospital, providing a lightweight and easily promotable in-hospital intelligent screening tool for ZNS TDM in children. It can assist clinicians in early identification of high-risk populations with abnormal concentrations, guide targeted plasma concentration retesting and individualized dose adjustment, enhance TDM monitoring efficiency, and optimize *in vivo* drug exposure levels. However, it must be objectively noted that this model only predicts the binary outcome of concentration target attainment and does not estimate precise plasma drug concentrations. The association between concentration target attainment status and clinical endpoints such as seizure freedom, adverse reactions, and treatment discontinuation has not yet been verified. The value of the model in improving clinical outcomes remains to be confirmed by future standardized prospective cohorts. In clinical practice, treatment efficacy should not be judged solely based on the model; dosing regimens should still be comprehensively adjusted in conjunction with the patient’s seizure control and tolerance. At present, the model lacks cross-center independent external validation; caution is warranted when extrapolating to patients in other medical institutions. Future work will involve multi-center independent external validation and supplement the analysis of the association between concentration stratification and long-term clinical epilepsy outcomes to complete the comprehensive evidence-based support system from target attainment risk prediction to clinical benefit.

## 5 Conclusion

Through rigorous multidimensional screening, this study identified dosage, age, gender, uric acid, total bilirubin, aspartate aminotransferase, and vitamin D as key factors influencing the attainment of therapeutic ZNS concentrations in pediatric epilepsy patients. The binary CART prediction model, constructed based on the aforementioned routine clinical indicators, demonstrated good discrimination, stable calibration, and clinical decision-making value in the pediatric population of this center, as verified by both the internal test set and the TSIV set.

By virtue of their robust capabilities in nonlinear data mining, multifactor integration, and rapid screening, machine learning algorithms effectively compensate for the limitations of traditional empirical prescribing and classical compartmental pharmacokinetic models in large-scale, single-center risk screening within real-world settings. These algorithms enable the rapid and convenient identification of pediatric patients at high risk for subtherapeutic ZNS concentrations, thereby providing intelligent tools to facilitate standardized TDM management, early intervention, and stratified individualized medication therapy for childhood epilepsy. Consequently, this approach demonstrates promising prospects for clinical translation in single-center scenarios.

It must be objectively acknowledged that classical pharmacokinetic models, represented by compartmental models, possess unique advantages in dynamic concentration simulation, multidimensional pharmacokinetic parameter quantification, and dose titration timing. They can predict drug concentrations, AUC, and metabolic time-effect indicators at arbitrary time points, serving as a crucial support for pharmacological mechanism research and precision quantitative dosing. The two modeling approaches possess distinct advantages, are irreplaceable, and are highly complementary: the machine learning model constructed in this study can screen key covariates and optimize variable combinations for subsequent population pharmacokinetic studies, while compartmental pharmacokinetic models can provide refined dose optimization evidence for high-risk patients. Future research should focus on integrating machine learning with pharmacokinetic modeling strategies, combined with multi-center prospective data, to refine multidimensional prediction systems, thereby providing a more comprehensive theoretical basis and technical support for the long-term precise and safe use of medication in children with epilepsy.

## Disclosure

The authors have no relevant affiliations or financial involvement with any organization or entity with a financial interest in or financial conflict with the subject matter or materials discussed in the manuscript. This includes employment, consultancies, honoraria, stock ownership or options, expert testimony, grants or patents received or pending, or royalties.

## Data Availability

The original contributions presented in the study are included in the article/Supplementary Material, further inquiries can be directed to the corresponding author.
