# Supplementary material for: Therapeutic drug monitoring of zonisamide in children with epilepsy: development and validation of a precise binary prediction model
Source: Front Pharmacol. 2026 Aug 27;17:1841966. doi: 10.3389/fphar.2026.1841966 (PMC13558151; doi:10.3389/fphar.2026.1841966)
Supplement: Supplementary file 1 [file Table1.docx]

**Table S1 Summary of missing data for each variable and distribution types for missing value imputation**

| **Variable** | **Dataset** | **Total sample**  **(N)** | **Missing sample**  **（N）** | **Missing rate**  **(%)** |
| --- | --- | --- | --- | --- |
| ALT(U/L) | Training cohort | 372 | 11 | 2.96 |
|  | Testing cohort | 159 | 5 | 3.14 |
| AST(U/L) | Training cohort | 372 | 11 | 2.96 |
|  | Testing cohort | 159 | 5 | 3.14 |
| UA(μmol/L) | Testing cohort | 159 | 9 | 5.66 |
|  | TSIV cohort | 431 | 23 | 5.34 |
| BUN(mmol/L) | Testing cohort | 159 | 9 | 5.66 |
|  | TSIV cohort | 431 | 23 | 5.34 |
| SCr(μmol/L) | Testing cohort | 159 | 9 | 5.66 |
|  | TSIV cohort | 431 | 23 | 5.34 |
| GFR(mL/(min·1.73m^2^)) | Testing cohort | 159 | 9 | 5.66 |
|  | TSIV cohort | 431 | 23 | 5.34 |
| VitD(nmol/L) | TSIV cohort | 431 | 17 | 3.94 |

Note: All continuous variables in this study failed to satisfy the parametric distribution assumptions for normality and log-normality; consequently, non-parametric empirical cumulative distribution random sampling probability imputation based on complete samples from the training set was uniformly adopted. Distribution parameters / empirical distributions were constructed exclusively using complete data from the training set; the test set and external validation set did not participate in distribution fitting. The imputation rules determined by the training set were consistently reused throughout the process to circumvent data leakage. Abbreviations: ALT: alanine aminotransferase; AST: aspartate aminotransferase; UA: uric acid; BUN: blood urea nitrogen; SCr: serum creatinine; GFR: glomerular filtration rate; VitD: vitamin D.Time-Split Internal Validation (TSIV).
