# Supplementary material for: Therapeutic drug monitoring of zonisamide in children with epilepsy: development and validation of a precise binary prediction model
Source: Front Pharmacol. 2026 Aug 27;17:1841966. doi: 10.3389/fphar.2026.1841966 (PMC13558151; doi:10.3389/fphar.2026.1841966)
Supplement: Supplementary file 2 [file Table2.docx]

**Table S2 Statistics of IQR outlier detection and truncated samples for continuous variables across datasets**

| **Variable** | **Dataset** | **Total sample**  **(N)** | **IQR outlier count（N）** | **Outlier**  **(%)** | **Censored**  **(N)** | **Group censoring**  **(%)** |
| --- | --- | --- | --- | --- | --- | --- |
| ALT(U/L) | Training cohort | 372 | 8 | 2.15 | 8 | 2.15 |
| AST(U/L) | Training cohort | 372 | 11 | 2.96 | 11 | 2.96 |
| UA(μmol/L) | Training cohort | 372 | 3 | 0.81 | 3 | 0.81 |
| BUN(mmol/L) | Training cohort | 372 | 9 | 2.42 | 9 | 2.42 |
| ALB(g/L) | Training cohort | 372 | 3 | 0.81 | 3 | 0.81 |
| WBC(10^9^/L) | Testing cohort | 159 | 6 | 3.77 | 6 | 3.77 |
| RBC(10^12^/L) | Testing cohort | 159 | 7 | 4.40 | 7 | 4.40 |
| TBIL(μmol/L) | TSIV cohort | 431 | 5 | 1.16 | 5 | 1.16 |
| PLT(%) | TSIV cohort | 431 | 16 | 3.71 | 16 | 3.71 |
| WBC(10^9^/L) | TSIV cohort | 431 | 12 | 2.78 | 12 | 2.78 |

Note: The IQR truncation thresholds were calculated exclusively from the training set and uniformly applied to all three datasets. Values falling below the lower limit of Q_1_ - 1.5 × IQR or exceeding the upper limit of Q_3_ + 1.5 × IQR were subjected to winsorization. All detected outlier samples underwent truncation; therefore, the number of outliers equals the number of truncated cases. Abbreviations: ALT: alanine aminotransferase; AST: aspartate aminotransferase; TBIL: total bilirubin;ALB: albumin; UA: uric acid; BUN: blood urea nitrogen; WBC: white blood cell; RBC: red blood cell; HPLT: platelet; Time-Split Internal Validation (TSIV).
