# Supplementary material for: Therapeutic drug monitoring of zonisamide in children with epilepsy: development and validation of a precise binary prediction model
Source: Front Pharmacol. 2026 Aug 27;17:1841966. doi: 10.3389/fphar.2026.1841966 (PMC13558151; doi:10.3389/fphar.2026.1841966)
Supplement: Supplementary file 3 [file Table3.docx]

**Table S3 Summary of original candidate variables and modeling selected variables, and theoretical basis for inclusion**

| **Variable** | **Initial Inclusion** | **Final Inclusion** | **Selection Rationale** | **Theoretical basis for inclusion** |
| --- | --- | --- | --- | --- |
| Gender  (male/female) | Yes | Yes | Determined by integrated feature screening | Gender may induce subtle differences in body composition, endocrine hormone levels, and hepatic enzyme activity in children, potentially influencing the distribution, metabolism, and clearance of zonisamide. Consequently, gender is considered a routine baseline confounding variable in individualized pharmacokinetic analyses. |
| Concomitant  (0/1) | Yes | No | Excluded by Univariate analysis | Concomitant can trigger drug-drug interactions through mechanisms such as competition for plasma protein binding, modulation of hepatic CYP450 enzyme activity, and alteration of organ perfusion and systemic homeostasis. These interactions modify the metabolic clearance rate of zonisamide and represent significant clinical confounding factors affecting its plasma concentration. |
| Weight  (kg) | Yes | No | Excluded by multicollinearity diagnosis | Weight serves as a core basis for individualized dosing in pediatrics. It is directly correlated with the apparent volume of distribution of zonisamide and significantly affects in vivo drug exposure and steady-state plasma concentrations, making it an indispensable baseline indicator in pediatric pharmacokinetic studies. |
| Age  (years) | Yes | No | Excluded by Lasso analysis | The maturation of hepatic and renal function, along with the activity of hepatic drug-metabolizing enzymes, develops progressively with age in children. These processes directly influence the hepatic metabolism and renal excretion efficiency of zonisamide, serving as core foundational covariates for the individualized prediction of antiepileptic drug plasma concentrations and significantly affecting in vivo drug disposition. |
| Dose  (mg/(kg・d)) | Yes | Yes | Determined by integrated feature screening | Dose is a direct and core determinant of plasma concentration. Zonisamide exhibits linear pharmacokinetic characteristics within the therapeutic window, and the daily dose demonstrates a significant correlation with steady-state plasma concentration, establishing it as a core independent variable for drug concentration prediction. |
| Duration  (Months) | Yes | No | Excluded by Univariate analysis | Duration use may induce adaptive physiological changes. Furthermore, the progression of chronic epilepsy over the course of treatment can indirectly affect hepatic and renal function as well as metabolic homeostasis, thereby influencing the stability of zonisamide plasma concentrations. |
| ALT  (U/L) | Yes | No | Excluded by Univariate analysis | As core indicators of hepatic function impairment, since zonisamide is primarily metabolized by the liver, hepatocellular injury can directly reduce hepatic drug-metabolizing capacity, leading to drug accumulation and abnormally elevated plasma concentrations. |
| AST  (U/L) | Yes | Yes | Determined by integrated feature screening | Reflecting the status of hepatocytes and systemic tissue injury, hepatic dysfunction and tissue damage can significantly interfere with the hepatic metabolic pathways of zonisamide and alter the drug clearance rate. |
| TBIL  (μmol/L) | Yes | Yes | Determined by integrated feature screening | Reflecting bilirubin metabolism and excretory function, impaired hepatic excretion is often accompanied by a decline in drug metabolic function, thereby affecting the clearance process of zonisamide. |
| TP  (g/L) | Yes | No | Excluded by Univariate analysis | Indicative of hepatic synthetic function and systemic nutritional status, TP levels can affect the plasma protein binding rate of the drug, altering the concentration of free zonisamide and its pharmacodynamic and metabolic characteristics. |
| ALB  (g/L) | Yes | No | Excluded by Univariate analysis | Zonisamide binds to plasma albumin; thus, ALB levels directly influence the drug's plasma protein binding rate and regulate free drug concentration, serving as a key biochemical indicator affecting pharmacokinetics. |
| UA  (μmol/L) | Yes | Yes | Determined by integrated feature screening | Indicative of renal metabolic excretion function and purine metabolism status, renal dysfunction can indirectly interfere with drug excretion processes and affect the in vivo metabolic balance of zonisamide. |
| BUN  (mmol/L) | Yes | No | Excluded by Univariate analysis | Reflecting renal excretory function and protein metabolism status, elevated BUN in cases of renal impairment is accompanied by decreased renal clearance, affecting the steady-state plasma concentration of zonisamide. |
| SCr  (μmol/L) | Yes | No | Excluded by multicollinearity diagnosis | As a core indicator of renal function, since zonisamide is partially excreted unchanged by the kidneys, renal impairment can reduce renal clearance, resulting in systemic drug accumulation. |
| GFR  (mL/(min·1.73m^2^)) | Yes | No | Excluded by multicollinearity diagnosis | Precisely reflecting glomerular filtration function, this indicator directly determines the renal excretion efficiency of zonisamide and serves as a core quantitative metric for assessing the impact of renal function on drug metabolism. |
| FBG  (mmol/L) | Yes | No | Excluded by Univariate analysis | Reflecting glucose metabolism homeostasis, glucose metabolic disorders can affect hepatic and renal blood perfusion and cellular metabolic function, indirectly modulating the in vivo metabolism and clearance of zonisamide. |
| WBC  (10^9^/L) | Yes | No | Excluded by Univariate analysis | Indicative of the body's inflammatory and immune status, chronic epilepsy and long-term antiepileptic drug use may induce a low-grade inflammatory response, indirectly affecting hepatic and renal metabolic functions and drug disposition processes. |
| RBC  (10^12^/L) | Yes | No | Excluded by Univariate analysis | Reflecting hematopoietic function and systemic nutritional status, children with chronic epilepsy on long-term medication are prone to nutritional and metabolic abnormalities. Systemic disturbances can indirectly influence the in vivo distribution and metabolic efficiency of the drug. |
| HGB  (g/L) | Yes | No | Excluded by Univariate analysis | Assessing anemia and oxygen supply status, anemia can lead to abnormalities in hepatic and renal perfusion and tissue oxygen metabolism, thereby interfering with the hepatic metabolism and renal excretion of zonisamide. |
| HCT  (10^9^/L) | Yes | No | Excluded by Univariate analysis | Reflecting concentration and blood cell ratios, these indicators indirectly represent systemic circulatory perfusion and influence the in vivo distribution and pharmacokinetic characteristics of the drug. |
| PLT  (%) | Yes | No | Excluded by Univariate analysis | Indicative of coagulation status and systemic stress, physiological stress and organ dysfunction resulting from long-term antiepileptic therapy can indirectly affect drug metabolic homeostasis. |
| VitD  (nmol/L) | Yes | Yes | Determined by integrated feature screening | VitD participates in regulating immune, inflammatory, and organ metabolic functions. VitD deficiency is prevalent in children with chronic epilepsy and may indirectly affect drug-metabolizing enzyme activity and pharmacokinetic characteristics. |

Note: Abbreviations: ALT: alanine aminotransferase; AST: aspartate aminotransferase; TBIL: total bilirubin; TP: total protein; ALB: albumin; UA: uric acid; BUN: blood urea nitrogen; SCr: serum creatinine; GFR: glomerular filtration rate; FBG: fasting blood glucose; WBC: white blood cell; RBC: red blood cell; HGB: hemoglobin; HCT: hematocrit; PLT: platelet; VitD: vitamin D.
